# Serum screening with Down's syndrome markers to predict pre-eclampsia and small for gestational age: Systematic review and meta-analysis

**DOI:** 10.1186/1471-2393-8-33

**Published:** 2008-08-04

**Authors:** Rachel K Morris, Jeltsje S Cnossen, Marloes Langejans, Stephen C Robson, Jos Kleijnen, Gerben ter Riet, Ben W Mol, Joris AM van der Post, Khalid S Khan

**Affiliations:** 1 Academic Department of Obstetrics and Gynaecology, University of Birmingham, Birmingham Women's Hospital, Birmingham, B15 2TG, UK

## Abstract

**Background:**

Reliable antenatal identification of pre-eclampsia and small for gestational age is crucial to judicious allocation of monitoring resources and use of preventative treatment with the prospect of improving maternal/perinatal outcome. The purpose of this systematic review was to determine the accuracy of five serum analytes used in Down's serum screening for prediction of pre-eclampsia and/or small for gestational age.

**Methods:**

The data sources included Medline, Embase, Cochrane library, Medion (inception to February 2007), hand searching of relevant journals, reference list checking of included articles, contact with experts. Two reviewers independently selected the articles in which the accuracy of an analyte used in Downs's serum screening before the 25^th ^gestational week was associated with the occurrence of pre-eclampsia and/or small for gestational age without language restrictions. Two authors independently extracted data on study characteristics, quality and results.

**Results:**

Five serum screening markers were evaluated. 44 studies, testing 169,637 pregnant women (4376 pre-eclampsia cases) and 86 studies, testing 382,005 women (20,339 fetal growth restriction cases) met the selection criteria. The results showed low predictive accuracy overall. For pre-eclampsia the best predictor was inhibin A>2.79MoM positive likelihood ratio 19.52 (8.33,45.79) and negative likelihood ratio 0.30 (0.13,0.68) (single study). For small for gestational age it was AFP>2.0MoM to predict birth weight < 10^th ^centile with birth < 37 weeks positive likelihood ratio 27.96 (8.02,97.48) and negative likelihood ratio 0.78 (0.55,1.11) (single study). A potential clinical application using aspirin as a treatment is given as an example.

There were methodological and reporting limitations in the included studies thus studies were heterogeneous giving pooled results with wide confidence intervals.

**Conclusion:**

Down's serum screening analytes have low predictive accuracy for pre-eclampsia and small for gestational age. They may be a useful means of risk assessment or of use in prediction when combined with other tests.

## Background

Pre-eclampsia (PET) and small for gestational age (SGA) remain significant causes of perinatal death and childhood disability [1-3]. PET has significant health implications for the mother with complications including adult respiratory distress syndrome, coagulopathy, renal and liver failure and stroke. Babies affected by SGA on reaching adulthood are at greater risk of developing cardiovascular disease, hypertension, and non-insulin dependent diabetes [4,5]. Both PET and SGA are characterized by a failure of the trophoblast invasion (at 16–22 weeks) into the spiral arteries.

Second trimester serum screening for Down's syndrome is routinely offered to women in the United Kingdom and United States, either with the triple test (alpha-fetoprotein (AFP), human chorionic gonadotrophin (HCG) and unconjugated estriol) or with the addition of inhibin A as the quadruple test. More recently first trimester screening with fetal nuchal translucency, HCG and pregnancy associated plasma protein A (PAPP-A) has provided an earlier, more effective screening method [6]. Due to their origin and sites of metabolism these biochemical markers may be useful in the prediction of PET and SGA, there are however conflicting reports in the literature. Maternal serum levels of these analytes have been shown to be associated with adverse outcome [7,8] with low levels of PAPP-A having been suggested as a marker for impaired placental function and placentation [9]. There are studies however reporting contrasting views [10].

Reliable antenatal identification of PET and SGA is crucial to judicious allocation of monitoring resources and use of preventative treatment [11] with the prospect of improving maternal and perinatal outcome. The variation in the design of research on accuracy of tests for prediction of PET and SGA, the scatter of this research across many databases and languages, and the dearth of clear collated up-to-date summaries of this literature contribute to the uncertainty about the best screening and monitoring strategies [12]. Systematic reviews of the literature can improve our ability to identify those pregnancies at increased risk of developing PET and SGA making additional use of test results already obtained for Down syndrome screening.

The purpose of our review was to investigate the accuracy of serum biochemical markers used in first and second trimester Down syndrome serum screening in predicting PET and/or SGA. We systematically reviewed the available literature and meta-analysed the data.

## Methods

The systematic review was based on our previously published prospective protocols [13,14] designed using widely recommended methods [15-18]. The protocols are available as Additional files 1 and 2.

### Data sources and searches

Electronic searches were performed by experienced clinical librarians targeting the prediction of PET and SGA. We searched Medline, Embase, the Cochrane Library (2006;4) and Medion from inception until February 2007. The search strategies are detailed in the published protocols [13,14] and in Additional file 3. The reference lists of all included primary and review articles were examined to identify cited articles not captured by electronic searches. No language restrictions were applied.

### Study selection

The first stage of study selection was the scrutinizing of the database by two reviewers to identify articles from title and/or abstract. In a second stage, a search based on keywords for each of the analytes under review was performed within the Reference Manager database. The results of this search were scrutinized by a second reviewer. In the final stage of study selection the full papers of identified articles were obtained with final inclusion or exclusion decisions made after independent and duplicate examination of the papers. We included studies that reported on singleton pregnancies at any level of risk in any healthcare setting using any serum biochemical test used in Down syndrome serum screening before the 25^th ^week of gestation. Test accuracy studies allowing generation of 2 × 2 tables were included.

### Data extraction and Study Quality Assessment

Further details on inclusion and exclusion criteria and extracted clinical, methodological and statistical data can be found in the published protocols.

Acceptable reference standards for PET were: persistent systolic blood pressure (SBP) ≥ 140 mmHg or diastolic blood pressure (DBP) ≥ 90 mmHg with proteinuria ≥ 0.3 g/24 hours or ≥ 1+ dipstick (= 30 mg/dl in a single urine sample), new after 20 weeks of gestation. Severe PET was defined as SBP ≥ 160 mmHg or DBP ≥ 110 mmHg with proteinuria ≥ 2.0 g/24 hours or ≥ 3+ dipstick, or of early onset < 34 weeks gestation. Superimposed PET was defined as the development of proteinuria ≥ 0.3 g/24 hours or ≥ 1+ dipstick after 20 weeks gestation in chronically hypertensive patients [19]. Acceptable reference standards for SGA included birth weight < 10^th ^centile adjusted for gestational age and based on local population values and absolute birth weight threshold < 2500 g. Severe SGA was defined as birth weight < 5^th ^or < 3^rd ^centile or < 1750 g or and preterm SGA for SGA leading to delivery < 37 weeks. Neonatal ponderal index < 10^th ^centile, skin fold thickness, and mid-arm circumference/head circumference were also assessed [20-24].

Disagreements were resolved by consensus or arbitration of a third reviewer. For multiple/duplicate publication of the same data set, the most recent and/or complete study was included only.

All included manuscripts were assessed by at least one reviewer for study and reporting quality using validated tools [25-30]. Methodological quality was defined as the confidence that the study design, conduct and analysis have minimized biases in addressing the research question, thereby focusing on the internal validity (i.e. the degree to which the results of an observation are correct for the patients being studied). Items considered important for a good quality paper were prospective design with consecutive recruitment, full verification of the test result with reference standard (> 90%), adequate description of the index test and use of appropriate reference standard, and application of any preventative treatments. Additional quality items were assessed for SGA papers; whether they excluded cases of PET from the results, whether fetuses with chromosomal and structural anomalies were excluded and whether stillbirths and intrauterine deaths were excluded from the results. Further explanation of the quality assessment can be found in Additional file 4.

We excluded from the statistical analysis any paper with a case-control design as this type of design in diagnostic test accuracy studies has been shown to be associated with bias and over/under estimation of accuracy [29].

### Data synthesis and Analysis

From the 2 × 2 tables the following were calculated with their 95% confidence intervals for individual studies; sensitivity (true positive rate), specificity (true negative rate) and the likelihood ratios (LR, the ratio of the probability of the specific test result in people who do have the disease to the probability in people who do not). LRs indicate by how much a given test result raises or lowers the probability of having the disease and have been recommended by Evidence-based Medicine Groups [31,32]. Results were pooled among groups of studies with similar characteristics, the same threshold for the index test (PET and SGA), same reference standard threshold for (SGA) and the same trimester for testing. Where 2 × 2 tables contained zero cells, 0.5 was added to each cell to enable calculations.

Sub-groups were defined at the start of the review based on clinical criteria known to affect prognosis, method of index test or study quality: level of risk of population (high or low based on authors assessment and calculated incidence rates from results); type of assay used for index test; whether babies with chromosomal anomalies were excluded from the results; use of preventative treatment; quality of study. Sub-group analyses were performed where there were at least 3 studies with similar characteristics within that group.

Heterogeneity was assessed graphically by looking at the distribution of the sensitivities and specificities in the receiver operating characteristic (ROC) space and LRs as a measurement of accuracy size using a Forest plot. The loglikelihood and X^2 ^test were used to assess for heterogeneity statistically. When X^2 ^p value > 0.05 (homogenous data) the fixed effect pooling method was used; where there was heterogeneity random effects pooling was used. Summary ROC plots were produced (data not shown). Sensitivity analysis was performed to check the robustness of our results. A p value of < 0.05 was used throughout for statistical significance.

All statistical analyses were performed using Meta-Disc software  and Statsdirect for drawing the Forest plots.

### Clinical application

The clinical impact of estimates of accuracy for a screening test depend on how the results of the test alter the patient's pre-test probability of disease, based on disease prevalence. The post-test probability can then be combined with estimates of effectiveness for known treatments [33]. From this data we can then calculate the number of women needed to be tested (number needed to test- NNTest), using a particular serum marker, to prevent one case of SGA with a particular treatment and the number needed to treat (NNTreat), the number of test positive women needed to be treated to prevent one case of SGA. In this review clinical application will be assessed using aspirin as this is the only treatment with any level of effectiveness for PET and SGA [11,34].

## Results

### Literature identification, study characteristics, and quality

**Figure 1:**
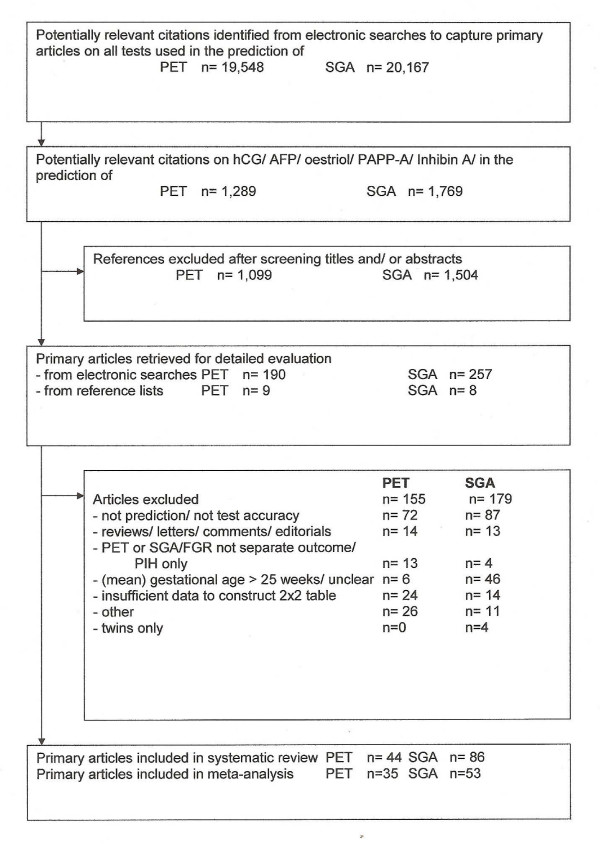
**Process from initial search to final inclusion for biochemical screening to predict pre-eclampsia/small for gestational age (up to February 2007).** PET preeclampsia; PIH pregnancy induced hypertension; SGA small for gestational age.

Figure 1 summarises the process of literature identification and selection. Tables detailing the individual study characteristics of the included studies are available in Additional file 5. There were twenty studies that reported on both PET and SGA.

### Pre-eclampsia

There were 44 included studies for pre-eclampsia [7,9,35-75] reporting on 169,637 women (4376 preeclamptic women, incidence 2.6%). Among these 44 studies, there were 35 cohort studies and nine case-control studies [41,43,44,48,51,55,63,72,73]. There were nine prospective studies, 10 retrospective and 25 were unclearly designed. Calculated incidence rates of PET ranged from 0.6–44%. Incidence rates of PET correlated poorly with descriptions of "high" or "low" risk study populations. Four of the studies were in "high-risk" populations (one in IVF patients, one in patients with abnormal uterine artery Doppler and two in patients with chronic hypertension) and in three of these studies the incidence of PET was > 4%. However in 15 of the "low-risk" studies the incidence was > 4% and in one study in which the inclusion criteria were unclearly reported. The remaining 25 studies were in low risk, screening populations with a calculated incidence of PET < 4%.

Ten studies were performed in the first trimester, 32 studies at a mean gestation between 15 to 20 weeks and two studies 20 to 24 weeks.

**Figure 2:**
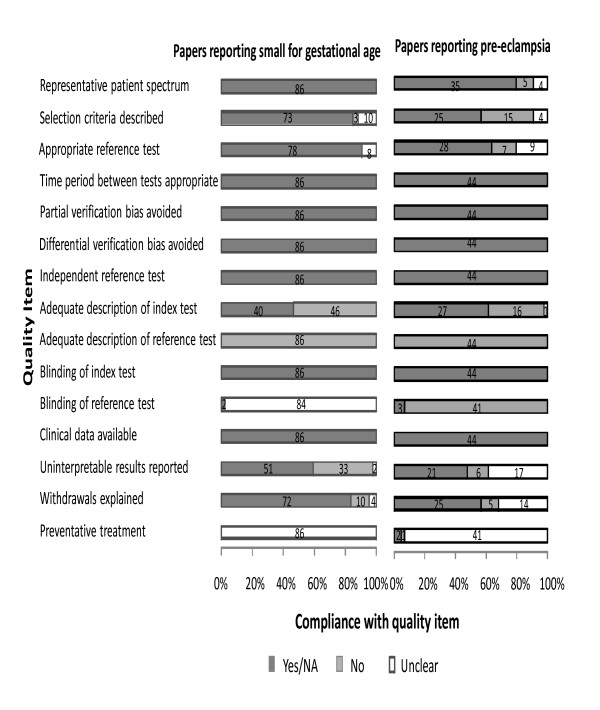
Bar chart showing quality of evidence on biochemical screening markers to predict small for gestational age and pre-eclampsia.

The quality assessment of included studies for PET is summarized in Figure 2. There was poor reporting of patient selection criteria, description of index and reference tests and blinding of the reference test. Only two studies reported clearly whether preventative treatment had been used. The nine case control studies were excluded from the final meta-analysis, leaving 35 cohort studies for analysis.

### Small for gestational age

There were 86 included studies for SGA [7,9,37,39,47,51,53-55,57,59-61,64-67,69,74,76-141], reporting on 382,005 women (20339 cases of SGA, incidence 5.32%). Among these studies, there were 61 cohort studies and 25 case control studies [53,55,76,77,82-85,88,89,94,96,97,104,113,116,119,123-125,130,131,133,135,140]. Thirty-one studies were prospective, 17 retrospective and 38 of unclear design. Calculated incidence rates of SGA correlated well with the threshold used in 78 of studies and poorly in 8, incidence range for birth weight < 10^th ^centile was 1.2–63%. Three of the studies were performed in high risk populations, whereas the remainder were performed in low risk or screening populations. Due to the inclusion criteria of the studies the majority of tests were performed between 15 to 20 weeks. There were ten studies reporting on first trimester screening. Fifty studies reported on birth weight < 10^th ^centile, 13 on birth weight < 5^th ^centile, 27 on birth weight < 2500 g, 1 on birth weight < 1500 g, 1 on birth weight < 15^th ^centile and 12 reported no threshold.

The quality assessment of included studies for SGA revealed deficiencies (Figure 2). Only 40 studies contained an adequate description of the performance of the index test. None of the studies reported clearly on the performance of the reference standard. Blinding of the reference test was also poorly reported as was the use of any treatment in between the index test and reference standard. These items of quality of study design are important in diagnostic accuracy reviews.

Four papers only distinguished between SGA with PET and SGA alone; intrauterine deaths and stillbirths were excluded from the results for SGA in only 16 papers, in the remainder it was unclear; chromosomal and structural anomalies were excluded from 62 studies, unclear in 24

Twenty-five case control studies and eight studies [78,81,98,105,122,127,129,138] in which thresholds for SGA were not defined were excluded from the final meta-analysis, leaving 53 studies.

### Data analysis

For both analysis for PET and analysis for SGA, there was significant heterogeneity in all results. As a consequence of this the random effects model was used throughout the study.

### Maternal serum alpha fetoprotein (AFP)

**Figure 3:**
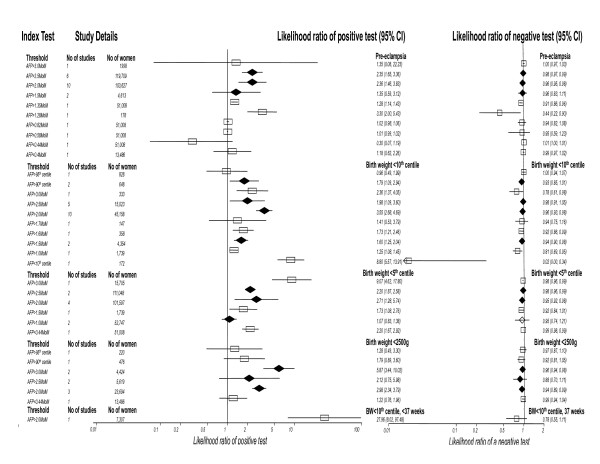
**Forest Plot showing likelihood ratio of a positive and negative test result with 95% confidence intervals (95% CI) for studies of alpha feto-protein (AFP) to predict pre-eclampsia and small for gestational age (birth weight threshold as indicated).** Results with diamonds are pooled results (number of studies as indicated), results with squares are single studies. The number of women included in the studies is shown, all studies second trimester testing.

The results for AFP are summarized in Figure 3, all studies were performed in the second trimester. For PET there were sixteen studies included in the meta-analysis. Thresholds that were most commonly used were > 2.0MoM (multiples of median) (10 studies) and > 2.5MoM (6 studies). The most accurate predictor was AFP>2.0 MoM; LR+ 2.36 (1.46,3.83), LR- 0.96 (0.95,0.98). (One study had a better positive LR however this threshold was chosen from receiver operating curve analysis AFP>1.28MoM; LR+ 3.30 (2.00,5.43), LR- 0.44(0.22,0.90)).

For SGA there were thirty studies included in the meta-analysis. The commonest threshold used were > 2.0MoM (10 studies) and > 2.5MoM (5 studies) to predict birth weight < 10^th ^centile. The best predictor for birth weight < 10^th ^centile was AFP<10^th ^centile; LR+ 8.80 (5.57,13.91), LR- 0.02 (0.00,0.34), this was a single study. For birth weight < 5^th ^centile and birth weight < 2500 g, AFP>3.0MoM was the most accurate predictor. The most accurate predictor overall was AFP>2.0MoM to predict severe SGA (birth weight < 10^th ^centile with birth < 37 weeks): LR+ 27.96 (8.02,97.48), LR- 0.78 (0.55, 1.11).

### Maternal serum human chorionic gonadotrophin (HCG)

**Figure 4:**
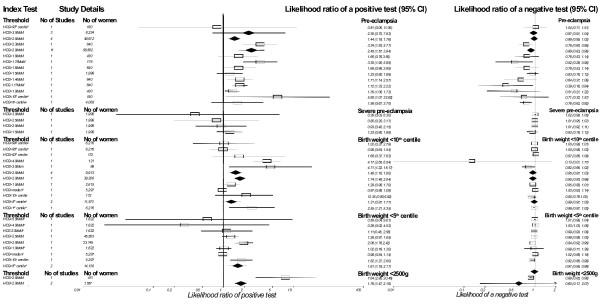
**Forest Plot showing likelihood ratio of a positive and negative test result with 95% confidence intervals (95% CI) for studies of human chorionic gonadotrophin (HCG) to predict pre-eclampsia and small for gestational age (birth weight threshold as indicated).** Results with diamonds are pooled results (number of studies as indicated), results with squares are single studies. The number of women included in the studies is shown. (^a ^first trimester testing).

The results for HCG are summarized in Figure 4. There were forty seven studies overall evaluating HCG, nine for free β-HCG, eight total β-HCG and 30 total HCG. For PET there were 21 included studies in the meta-analysis, 3 looked at testing in the first trimester. The commonest thresholds used were HCG>2.0MoM (12 studies), HCG>2.5MoM (4 studies) and HCG>3.0MoM (3 studies). The most accurate predictor was HCG>2.0MoM with second trimester testing; LR+ 2.45 (1.57,3.84), LR- 0.89 (0.83,0.96). There was one study looking at severe PET as the outcome, results showed no improvement in prediction.

For SGA there were 22 included studies in the meta-analysis, 5 looked at testing in the first trimester. The commonest thresholds used were HCG>2.0MoM (7 studies) and HCG>2.5MoM (4 studies) for birth weight < 10^th ^centile. The most accurate predictor for birth weight < 10^th ^centile was HCG>2.0MoM; LR+ 1.74 (1.48,2.04), LR- 0.95 (0.93,0.96). For birth weight < 5^th ^centile HCG>2.0MoM in the second trimester was the most accurate and for birth weight < 2500 g HCG>2.5MoM.

### Maternal serum unconjugated Estriol

**Figure 5:**
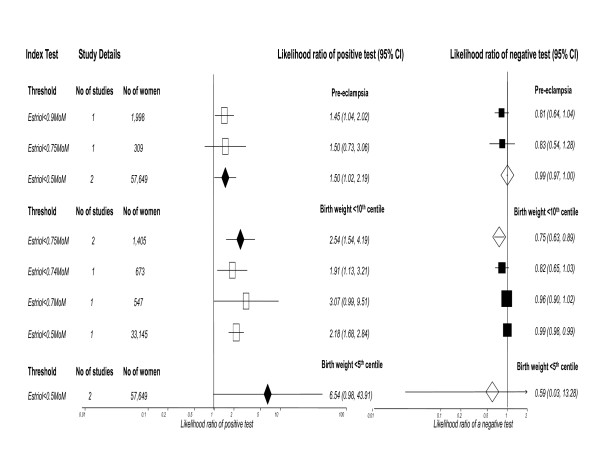
**Forest Plot showing likelihood ratio of a positive and negative test result with 95% confidence intervals (95% CI) for studies of estriol to predict pre-eclampsia and small for gestational age (birth weight threshold as indicated).** Results with diamonds are pooled results (number of studies as indicated), results with squares are single studies. The number of women included in the studies is shown, all studies second trimester testing.

The results for unconjugated estriol are summarized in Figure 5, all studies were performed in the second trimester. For PET there were 4 included studies, the commonest threshold being estriol<0.5MoM (2 studies), this was also the most accurate predictor; LR+ 1.50 (1.02,2.19), LR- 0.99 (0.97,1.00).

For SGA there were 7 included studies, the commonest threshold was estriol<0.75MoM (2 studies) for birth weight < 10^th ^centile. The most accurate predictor for birth weight < 10^th ^centile was estriol<0.75MoM; LR+ 2.54 (1.54,4.19), LR- 0.75 (0.63,0.89). For birth weight < 5^th ^centile there were 2 studies for estriol<0.5 MoM; LR+ 6.54 (0.98,43.91), LR- 0.59 (0.03,13.28).

### Maternal serum pregnancy associated plasma protein A (PAPP-A)

**Figure 6:**
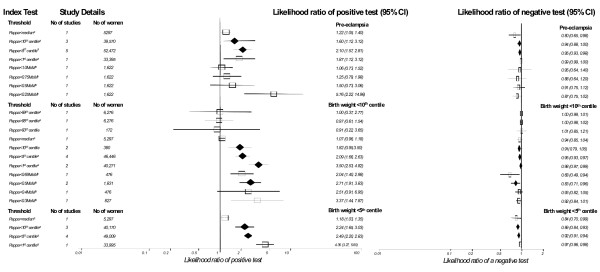
**Forest Plot showing likelihood ratio of a positive and negative test result with 95% confidence intervals (95% CI) for studies of pregnancy associated plasma protein A (PAPPA) to predict pre-eclampsia and small for gestational age (birth weight threshold as indicated).** Results with diamonds are pooled results (number of studies as indicated), results with squares are single studies. The number of women included in the studies is shown. (^a ^first trimester testing).

The results for PAPP-A are summarized in Figure 6. For PET there were 16 included studies, all performed in the first trimester, the commonest threshold was PAPP-A<5^th ^centile (5 studies) and PAPP-A<10^th ^centile (3 studies). The most accurate predictor was PAPP-A<5^th ^centile; LR+ 2.10 (1.57,2.81), LR- 0.95 (0.93,0.98).

For SGA there were 10 included studies, 7 were performed in the first trimester, the commonest thresholds were PAPP-A < 5^th ^centile (4 studies), PAPP-A<10^th ^centile (5 studies) for birth weight < 10^th ^centile. The most accurate predictor for birth weight < 10^th ^centile was PAPP-A<1^st ^centile; LR+ 3.50 (2.53,4.82), LR- 0.98 (0.97,0.99). For birth weight < 5^th ^centile, the most accurate predictor was again PAPP-A<1^st ^centile; LR+ 4.36 (3.27,5.80), LR- 0.97 (0.96,0.98).

### Maternal serum inhibin A

**Figure 7:**
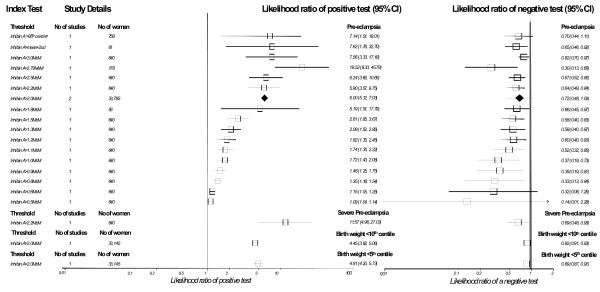
Forest Plot showing likelihood ratio of a positive and negative test result with 95% confidence intervals (95% CI) for studies of Inhibin A to predict pre-eclampsia and small for gestational age (birth weight threshold as indicated). Results with diamonds are pooled results (number of studies as indicated), results with squares are single studies. The number of women included in the studies is shown. (^a ^first trimester testing).

The results for inhibin A are summarized in Figure 7. For PET there were 6 included studies, 1 performed in the first trimester, the commonest threshold being inhibin A>2.0MoM (2 studies) with a LR+ 6.00 (5.12,7.03), LR- 0.72 (0.48,1.09). The most accurate predictor for PET was inhibin A>2.79MoM; LR+ 19.52 (8.33,45.79), LR- 0.30 (0.13,0.68), however this result was derived from one study using a receiver operating characteristic curve to determine threshold.

For SGA there was only one study, looking at second trimester testing, using a cut-off of inhibin A>2.0MoM, the results for prediction of birth weight < 10^th ^centile were LR+ 4.45 (3.92,5.06), LR- 0.92 (0.91,0.93) and birth weight < 5^th ^centile; LR+ 4.91 (4.20,5.73), LR- 0.89 (0.87,0.91).

### Triple test (serum AFP, HCG and unconjugated estriol)

There were no included studies for PET. For SGA there were 2 studies, second trimester testing, with different cut-offs for prediction of birth weight < 10^th ^centile: triple test > 1:190 LR+ 1.07 (0.60,1.91), LR- 0.98 (0.82,1.17) and triple test>1:250 LR+ 2.71 (1.77,4.17), LR- 1.19 (0.01,2.47).

### Gestation of testing

Table 1 shows the different results achieved where testing was performed in both the first and second trimester. Overall for HCG, testing in the second trimester was more accurate.

**Table 1:** Subgroup analyses of accuracy of biochemical screening to predict small for gestational age and pre-eclampsia (random effects pooling).

***Small for gestational age***				
***Analyte ****Subgroup*	***Positive Likelihood Ratio (95% CI)***	***Negative Likelihood Ratio (95% CI)***	***Sensitivity (95% CI)***	***Specificity (95% CI)***

***HCG>90^th ^centile (BW<10th centile)***				

*Trimester*				
First	1.48 (0.57–3.81)	0.92 (0.72–1.17)	0.21 (0.06–0.46)	0.86 (0.79–0.91)
Second	1.68 (0.37–7.63)	0.97 (0.86–1.09)	0.08 (0.01–0.26)	0.95 (0.90–0.98)

***HCG<10^th ^centile (BW<10th centile)***				

*Trimester*				
First	1.29 (0.05–33.56)	1.14 (0.53–2.43)	0.13 (0.10–0.16)	0.60 (0.57–0.63)
Second	2.35 (0.80–6.92)	0.90 (0.76–1.08)	0.16 (0.05–0.36)	0.93 (0.88–0.97)

***HCG>2.0MoM (BW<5th centile)***				

*Trimester*				
First	0.96 (0.55–1.68)	1.01 (0.88–1.17)	0.20 (0.10–0.34)	0.79 (0.77–0.81)
Second	2.08 (1.78–2.42)	0.94 (0.92–0.95)	0.12 (0.10–0.14)	0.94 (0.94–0.95)

***PAPPA<10^th ^centile (BW<10^th ^centile)***				
*Trimester*				
First	1.68 (1.25–2.27)	0.93 (0.88–0.98)	0.17 (0.16–0.19)	0.90 (0.89–0.90)
Second	1.82 (0.95–3.50)	0.91 (0.75–1.05)	0.20 (0.10–0.33)	0.89 (0.85–0.92)

***Pre-eclampsia***				

***HCG>2.0MoM***				

*Trimester*				
First	1.77 (1.07–2.92)	0.80 (0.60–1.07)	0.37 (0.19–0.58)	0.79 (0.77–0.81)
Second	2.45 (1.57–3.84)	0.89 (0.83–0.96)	0.19 (0.17–0.21)	0.93 (0.93–0.93)

(CI confidence intervals, AFP alpha feto-protein, HCG human chorionic gonadotrophin, BW birth weight, MoM multiple of median). Analyses according to gestation of testing of accuracy of biochemical screening to predict small for gestational age and pre-eclampsia (random effects pooling). (CI confidence intervals, HCG human chorionic gonadotrophin, BW birth weight, MoM multiple of median, PAPPA pregnancy associated plasma protein A)

### Sub-group and sensitivity analysis

For sub group analysis, a sub-group had to include at least three studies within each analyte and threshold and thus was only possible for calculated incidence of disease. The results for sub-group analysis are shown in Table 2. There was no significant difference between the subgroups.

**Table 2:** Subgroup analyses of accuracy of biochemical screening to predict small for gestational age and pre-eclampsia (random effects pooling)

***Small for gestational age***				
***Analyte ****Subgroup*	***Positive Likelihood Ratio (95% CI)***	***Negative Likelihood Ratio (95% CI)***	***Sensitivity (95% CI)***	***Specificity (95% CI)***

***AFP>2.0MoM (BW<10th centile)***				

*Incidence*				
>10%	2.69 (1.36–5.31)	0.98 (0.96–1.00)	0.04 (0.02–0.08)	0.98 (0.98–0.99)
≤ 10%	3.71 (2.66–5.16)	0.93 (0.88–0.97)	0.06 (0.05–0.07)	0.98 (0.98–0.98)

***HCG>2.0MoM(BW<10th centile)***				

*Incidence*				
>10%	1.53 (1.1–2.12)	0.89 (0.77–1.04)	0.29 (0.22–0.37)	0.79 (0.77–0.82)
≤ 10%	1.92 (1.72–2.13)	0.95 (0.94–0.96)	0.11 (0.1–0.12)	0.94 (0.94–0.95)

***Pre-eclampsia***				

***AFP>2.0MoM***				

*Incidence*				
>4%	0.85 (0.41–1.78)	1.01 (0.97–1.06)	0.06 (0.02–0.13)	0.93 (0.91–0.94)
≤ 4%	2.98 (1.77–5.03)	0.96 (0.95–0.97)	0.08 (0.06–0.09)	0.96 (0.96–0.96)

***HCG>2.0MoM***				

*Incidence*				
>4%	2.45 (0.65–2.93)	0.68 (0.42–1.1)	0.25 (0.21–0.3)	0.92 (0.91–0.93)
≤ 4%	2.36 (1.81–3.08)	0.89 (0.85–0.95)	0.18 (0.16–0.2)	0.93 (0.92–0.93)

(CI confidence intervals, AFP alpha feto-protein, HCG human chorionic gonadotrophin, BW birth weight, MoM multiple of median)

Most of the studies included in the review excluded fetuses with other structural or chromosomal anomalies from the results and included live births only thus subgroup analysis could not be performed in these areas. Sensitivity analysis including only those studies with these characteristics showed no significant difference. The same was true for the assessment of study quality i.e. most studies were of a similar quality to make sub-group analysis impossible but sensitivity analysis showed no difference when extremely low quality studies were excluded.

Forest plots of sensitivity and specificity are shown in Additional file 6. Summary receiver operating characteristic curves are available from the authors on request.

### Clinical application with aspirin

The results for clinical application with aspirin for SGA are shown in Table 3 and for PET in Table 4. The results show that by testing with inhibin A for PET or SGA in a low risk population we can reduce the number of women needed to treat to prevent one case of SGA from 90 to 30 and for PET from 323 to 27, having to test 909 and 469 women respectively.

**Table 3:** Serum screening among pregnant women and number of women needed to be tested and treated with aspirin to prevent one case of SGA (birth weight < 10^th ^centile).

**Test result**	**Prevalence SGA (%)**	**Probability of SGA after testing positive (%)**	Risk of SGA after treatment*	**Probability of SGA after treatment**	**NNTest^1^**	**NNTreat^2^**
No test, no treatment^3^	10.0	10.0	-	10.0	-	-
No test, treat all^3^	10.0	-	0.90	9.0	-	90

**Alpha feto-protein>2.0MoM: Sensitivity 60%; Specificity 98%**						
Test all, treat test positives	10.0	28.3	0.90	25.4	167	35

**Human chorionic gonadotrophin>2.0MoM: Sensitivity 12%; Specificity 94%**						
Test all, treat test positives	10.0	16.2	0.90	14.6	833	62
**Unconjugated estriol<0.75MoM: Sensitvity 37%; Specifcitiy 88%**						
Test all, treat test positives	10.0	22.0	0.90	19.8	270	45
**Pregnancy associated plasma protein A (PAPP-A)<1^st ^centile: Sensitivity 3%; Specificity 99%**						
Test all, treat test positives	10.0	28.0	0.90	25.2	3333	36
**Inhibin A>2.0MoM: Sensitivity 11%; Specificity 98%.**						
Test all, treat test positives	10.0	33.1	0.90	29.8	909	30
**Alpha feto-protein>2.0MoM to predict severe FGR: Sensitivity 22%, Specificity 99%**						
Test all, treat test positives	1.0	22.0	0.90	19.8	454	45

* RR 0.90 (95% CI 0.84–0.97) Askie et al. Antiplatelet agents for prevention of pre-eclampsia: meta-analysis of individual patient data. Lancet 2007;369:1791–98;^11^.

^1 ^NNTest is number needed to test and treat with aspirin to prevent one case of SGA calculated by 1/(proportion true positives (TP) – (proportion TP * RR)).

^2 ^NNTreat is number need to treat if only treat test positives with aspirin calculated by 1/(probability after testing positive – probability after treatment).

^3 ^Numbers are equal for all tests regardless of threshold, sensitivity and specificity.

MoM multiples of median

SGA small for gestational age

**Table 4:** Serum screening among pregnant women and number of women needed to be tested and treated with aspirin to prevent one case of PET.

**Test result**	**Prevalence PET (%)**	**Probability of PET after testing positive (%)**	Risk of PET after treatment*	**Probability of PET after treatment**	**NNTest^1^**	**NNTreat^2^**
No test, no treatment^3^	3.0	3.0	-	3.0	-	-
	10.0	10.0		10.0		
No test, treat all^3^	3.0	-	0.9	2.8	-	323
	10.0	-	0.9	9.0	-	90

**Alpha feto-protein>2.0MoM: Sensitivity 7%; Specificity 96%**						
Test all, treat test positives	3.0	7.3	0.9	6.1	4762	147
	10.0	26.2	0.9	18.6	1429	48

**Human chorionic gonadotrophin>2.0MoM, second trimester: Sensitivity 19%; Specificity 93%**						
Test all, treat test positives	3.0	7.5	0.9	6.3	1754	142
	10.0	27.2	0.9	19.3	526	47
**Unconjugated estriol<0.5MoM: Sensitvity 6%; Specifcitiy 96%**						
Test all, treat test positives	3.0	4.6	0.9	4.0	5556	226
	10.0	16.7	0.9	12.8	1667	70
**Pregnancy associated plasma protein A (PAPP-A)<5th centile: Sensitivity 9%; Specificity 95%**						
Test all, treat test positives	3.0	6.5	0.9	5.5	3704	167
	10.0	23.3	0.9	17.0	1111	53
**Inhibin A>2.79MoM: Sensitivity 71%; Specificity 96%.**						
Test all, treat test positives	3.0	6.0	0.9	3.4	469	27
	10.0	216.9	0.9	61.2	141	15

* RR 0.90 (95% CI 0.81–1.01) Askie et al. Antiplatelet agents for prevention of pre-eclampsia: a meta-analysis of individual patient data. Lancet 2007;369:1791–98^11^.

^1 ^NNTest is number needed to test and treat with aspirin to prevent one case of FGR calculated by 1/(proportion true positives (TP) – (proportion TP * RR)).

^2 ^NNTreat is number need to treat if only treat test positives with aspirin calculated by 1/(probability after testing positive – probability after treatment).

^3 ^Numbers are equal for all tests regardless of threshold, sensitivity and specificity.

MoM multiples of median

PET pre-eclampsia

## Discussion

We evaluated the accuracy of five serum screening markers used in Down's syndrome screening. The results showed low predictive accuracy overall. For PET the best predictor was inhibin A>2.79MoM. However, it is important to point out that this threshold was determined from a receiver operating characteristic curve and based on a single study. For SGA the best predictor overall for birth weight < 10^th ^centile was AFP<10^th ^centile while AFP>3.0MoM was the best predictor of birth weight < 5^th ^centile. These results were both based on single studies. AFP and inhibin A showed improvements in predictive accuracy when looking at severe disease for SGA and PET respectively. HCG showed improved prediction when comparing second trimester to first trimester testing.

The strength of our review and validity of its findings lies in the methodological strengths used. We complied with existing guidelines for the reporting of systematic reviews [18] and also guidelines specific to the reporting of systematic reviews of observational studies [142]. We performed extensive literature searches without language restrictions. We paid careful attention to assessment of quality of study design and reporting (The Quorum statement for this review is shown in Additional file 7).

Previously published reviews in this area are restricted to a systematic review evaluating predictive tests for PET [143]. This review concluded that the tests investigated had a low predictive value, the methodology of this review has however been criticized [144] and was restricted in the thresholds and tests it reviewed. To our knowledge there are no previously reported systematic reviews in this area for SGA.

We have primarily reported likelihood ratios in this review as they are thought to be more clinically meaningful than sensitivities and specificities, the use of likelihood ratios allowing us to determine post test probabilities of disease based on Bayes' theorem. Recent research suggests that independently pooled likelihood ratios should be interpreted with caution as positive and negative likelihood ratios are related statistics (just like sensitivity and specificity) [145]. We also pooled sensitivity and specificity and found no difference in the interpretation of the results. Bivariate meta-analysis is a new statistical technique that explicitly incorporates the correlation between sensitivity and specificity in a single model [146], its use is however not yet widespread nor is it easily interpreted.

Our assessment of study quality was hindered by lack of clear reporting, which is a common problem in diagnostic reviews as standards for quality and checklists for assessing it are fairly new. It has been previously reported that poor study design and conduct can affect the estimates of diagnostic accuracy [28,29] however, it is not entirely clear how individual aspects of quality may effect this and to what magnitude particularly in the area of Obstetrics. Application of quality scores has been shown to be of little value on diagnostic reviews [147] however, due to the lack of clear reporting it was not possible to perform sub-group analysis based on individual quality criteria.

One of the areas in which reporting was uniformly poor was in the details provided regarding performance of the reference standard. In PET definitions have changed over time with previous definitions including change increases in blood pressure. The measurement of blood pressure was poorly reported. It is important to record diastolic blood pressure with Korotkoff phase V as this is more reliably recorded and reflects true diastolic blood pressure [148-150]. For SGA there is still no convincing evidence as to which is the best definition of the condition at birth nor which is the best predictor of future infant and childhood morbidity and mortality for term infants. Population based birth weight standards were the most commonly used, however it is important to realize that these do not distinguish between the small healthy infant and the compromised infant. Customised growth charts that are adjusted for sex, gestation, parity, maternal weight and height and ethnicity, have been shown to improve the detection of infants at risk of stillbirth [151] while neonatal indices have been shown to identify the malnourished infant at risk of peripartum asphyxia [152]. Unfortunately these were rarely used as outcome measures in the included reviews.

Confounding factors in measurement of serum screening markers but mainly AFP is its association with intrauterine death, preterm labour and chromosomal and structural anomalies [54,57,60]. Ideally all the included papers in this review should have included only women with live births and fetuses with no other chromosomal or structural anomalies, this however was not always clearly reported. Sensitivity analysis, including only studies that did report exclusion of these subjects showed no significant difference in estimates of test accuracy.

In this review we have also assumed that the markers act independently but this may not be the case. The relationship between PET and SGA must also be taken into account. For HCG measurement the risk of SGA has been shown by logistic regression to be dependent on the presence of PET [99]. Ideally included cases of SGA for this review would have been those where there was no PET but this was again poorly reported.

When assessing the clinical relevance of these tests it is important to look at severe disease as this causes the majority of maternal, fetal and neonatal complications and thus prediction and prevention of this form of disease would have the greatest health impact. For the studies included in the meta-analysis there were only three that had results for either severe PET or SGA and these were insufficient to make an accurate assessment of the prediction of this form of disease.

The calculations of NNTreat and NNTest show that we can reduce the number of women needed to treat with aspirin to prevent one case of SGA/PET if we first test with a serum screening marker and then only treat the test positives. As aspirin is not routinely used as a treatment these calculations serve to contextualize the predictive value of these markers as individual tests. The costs of introducing aspirin as a treatment would need to be balanced against the costs of the test, costs of failing to treat the women with a false negative result that then go on to develop disease and any patient costs in terms of anxiety from screening and over treatment in the false positive category. To thus calculate the true clinical effectiveness of these tests these results would need to be incorporated in to a full cost-effectiveness analysis.

As PET and SGA are diseases with relatively low prevalence a clinically useful test would need to have a high positive LR (> 10) and low negative LR (< 0.10) [153]. From the results of this review it is unlikely that any one serum screening marker in isolation will provide this. Future research should thus concentrate in two areas. The first should be to address the limitations within the primary literature as identified by this review; poor reporting, exclusion of intrauterine deaths and chromosomal and structural anomalies from the results, separation of PET and SGA, prediction of severe disease. This may not necessarily require further primary research as there are sufficient large, well designed cohort studies available but meta-analysis based on individual patient data. Secondly future research should focus on combinations of markers as predictors and combinations of tests such as serum screening markers and uterine artery Doppler [154] to improve the predictive accuracy to a clinically useful value.

As Down's serum screening is routinely performed in many developed countries the cost of implementing use of these results as a predictive test for PET and SGA would be small. However as aspirin is the only preventative treatment with any proven benefit in these conditions and has minimal adverse events this cost has to be compared to that of implementing aspirin treatment to all pregnant women.

## Conclusion

Down's serum screening analytes have low predictive accuracy for pre-eclampsia and small for gestational age. They may be a useful means of risk assessment or of use in prediction when combined with other tests.

## Competing interests

The authors declare that they have no competing interests.

## Authors' contributions

The following authors were responsible for the study concept and design: RKM, JSC, GtR, BWM, JAMvdP, JK, KSK, SCR. The following authors were responsible for acquisition of data: RKM, JSC, ML, BWM.

Analysis and interpretation of data was performed by the following authors: RKM, JSC, KSK. Drafting of the manuscript was performed by: RKM, JSC, ML, GtR, BWM, JAMvdP, KSK, JK, SCR. Statistical analysis was performed by: RKM, JSC, KSK. All authors read and approved the final manuscript. 

RKM had full access to all of the data in the study and takes responsibility for the integrity of the data and the accuracy of the data analysis.

## Pre-publication history

The pre-publication history for this paper can be accessed here:



## Supplementary Material

Additional file 1 "Prediction of pre-eclampsia: a protocol for systematic reviews of test accuracy." Study protocol for pre-eclampsia systematic reviews. Click here for file

Additional file 2 "The value of predicting restriction of fetal growth and compromise of its wellbeing: Systematic quantitative overviews (meta-analysis) of test accuracy literature." Study protocol for fetal growth restriction systematic reviews. Click here for file

Additional file 3 "Search strategies for biochemical markers used in Down's serum screening to predict preeclampsia/small for gestational age." Electronic search strategies for systematic reviews. Click here for file

Additional file 4 "Guide to QUADAS for Down's syndrome markers to predict pre-eclampsia/small for gestational age." Guide to quality assessment of included papers in review using QUADAS tool. Click here for file

Additional file 5 "Study characteristics of studies of included studies for maternal serum biochemical (Down syndrome) screening to predict pre-eclampsia and small for gestational age." Click here for file

Additional file 6 "Forest plots of sensitivity and specificity." Results of sensitivity and specificity displayed as Forest plots. Click here for file

Additional file 7 "Improving the quality of reports of meta-analyses of randomised controlled trials: the Quorum Statement checklist." The Quorum statement checklist. Click here for file
