# Supplementary material for: Serum screening with Down's syndrome markers to predict pre-eclampsia and small for gestational age: Systematic review and meta-analysis
Source: BMC Pregnancy Childbirth. 2008 Aug 4;8:33. doi: 10.1186/1471-2393-8-33 (PMC2533288; doi:10.1186/1471-2393-8-33)
Supplement: Additional file 1 — "Prediction of pre-eclampsia: a protocol for systematic reviews of test accuracy." Study protocol for pre-eclampsia systematic reviews. [file 1471-2393-8-33-S1.pdf]

BMC Pregnancy and Childbirth

Study protocol

Open Access

Prediction of pre-eclampsia: a protocol for systematic reviews of test accuracy

Jeltsje S Cnossen*[1,2], Joris AM van der Post[2], Ben WJ Mol[2], Khalid S Khan[3], Catherine A Meads[4] and Gerben ter Riet[1,5]

Address: [1]Academic Medical Center-University of Amsterdam, Department of General Practice, PO Box 22660, 1100 DD, Amsterdam, The Netherlands, [2]Academic Medical Center-University of Amsterdam, Department of Obstetrics and Gynaecology, PO Box 22660, 1100 DD, Amsterdam, The Netherlands, [3]Department of Obstetrics and Gynaecology, Birmingham Women's Hospital/University of Birmingham, Birmingham, B15 2TG, UK, [4]Department of Public Health and Epidemiology, Public Health Building, University of Birmingham, Birmingham, B15 2TT, UK and [5]Horten Center, University of Zurich, Bolleystrasse 40, CH-8091, Zurich, Switzerland

Email: Jeltsje S Cnossen* - j.s.cnossen@amc.uva.nl; Joris AM van der Post - j.a.vanderpost@amc.uva.nl; Ben WJ Mol - b.w.mol@amc.uva.nl; Khalid S Khan - k.s.khan@bham.ac.uk; Catherine A Meads - c.a.meads@bham.ac.uk; Gerben ter Riet - g.terriet@amc.uva.nl

* Corresponding author

Published: 19 October 2006

BMC Pregnancy and Childbirth 2006, **6**:29 doi:10.1186/1471-2393-6-29

Received: 13 September 2006
Accepted: 19 October 2006

This article is available from: http://www.biomedcentral.com/1471-2393/6/29

© 2006 Cnossen et al; licensee BioMed Central Ltd.
This is an Open Access article distributed under the terms of the Creative Commons Attribution License (http://creativecommons.org/licenses/by/2.0), which permits unrestricted use, distribution, and reproduction in any medium, provided the original work is properly cited.

Abstract

Background: Pre-eclampsia, a syndrome of hypertension and proteinuria, is a major cause of maternal and perinatal morbidity and mortality. Accurate prediction of pre-eclampsia is important, since high risk women could benefit from intensive monitoring and preventive treatment. However, decision making is currently hampered due to lack of precise and up to date comprehensive evidence summaries on estimates of risk of developing pre-eclampsia.

Methods/Design: A series of systematic reviews and meta-analyses will be undertaken to determine, among women in early pregnancy, the accuracy of various tests (history, examinations and investigations) for predicting pre-eclampsia. We will search Medline, Embase, Cochrane Library, MEDION, citation lists of review articles and eligible primary articles and will contact experts in the field. Reviewers working independently will select studies, extract data, and assess study validity according to established criteria. Language restrictions will not be applied. Bivariate meta-analysis of sensitivity and specificity will be considered for tests whose studies allow generation of 2 × 2 tables.

Discussion: The results of the test accuracy reviews will be integrated with results of effectiveness reviews of preventive interventions to assess the impact of test-intervention combinations for prevention of pre-eclampsia.

Background

Pre-eclampsia belongs to a group of hypertensive disorders in pregnancy, that can be divided into gestational hypertension, chronic hypertension, pre-eclampsia, and pre-eclampsia superimposed on chronic hypertension. Hypertension is a common medical complication during pregnancy. It is usually defined as systolic blood pressure of at least 140 mmHg and/or diastolic blood pressure of at least 90 mmHg. Pre-eclampsia is defined as hypertension accompanied by proteinuria first detected after 20 weeks gestation. Proteinuria is defined as at least 300 mg protein in a 24 hour urine collection (or ≥ 1 + dipstick (30

mg/dL) in a single urine sample) [1-3]. In the past, other components such as oedema or a rise in systolic and/or diastolic blood pressure have been included in the definition of pre-eclampsia. However, these components do not define a group at risk of poor outcome[4,5]. Oedema is also a common feature of normal pregnancy. Women with mild pre-eclampsia generally have no symptoms. However, women with severe pre-eclampsia (usually RR ≥ 160/110 mmHg and/or proteinuria ≥ 2–5 g/24 hours) may have signs and symptoms such as renal insufficiency (reduced urinary volume, raised serum creatinine), liver disease (upper abdominal pain, elevated liver enzymes), neurological disturbances (headache, visual disturbances, exaggerated tendon reflexes, convulsions (eclampsia)), and haematological disturbances (thrombocytopenia, disseminated intravascular coagulation, haemolysis) [1-3].

The precise aetiology of pre-eclampsia is still unknown. Factors that appear to have a role include the placenta, maternal immune response, genetic predisposition, and maternal vascular disease [6]. The central cause of pre-eclampsia lies within the placenta, and resolution of pre-eclampsia starts with the removal of the placenta at delivery. Both abnormal implantation and excessive placental tissue have been implicated as the underlying pathology in pre-eclampsia [7,8]. Failure of the normal invasion of throphoblast cells leads to maladaptation of maternal spiral arterioles in the uterus, resulting in small diameter, high resistance blood vessels that are unable to meet the increasing demand for blood supply to the placenta [9]. On the other hand, reduced perfusion may exist in large normally implanted placentas, such as in multiple pregnancies. Implantation of the placenta and vascular changes are completed by 20–22 weeks gestation [9]. So although pre-eclampsia is usually diagnosed in the second half of pregnancy, damage has already occurred at an earlier stage of pregnancy. These changes may lead to alterations of products released from the placenta into the maternal circulation, which then may be used as early biochemical markers for disease.

Normal pregnancy requires adaptation of the maternal immune response, so that the foetus and placenta, being partly allogenic, are not rejected. In pre-eclampsia, this adaptation may be inadequate in a first pregnancy with a new partner (and limited sperm exposure), while in subsequent pregnancies with the same partner the risk is lower [6,10,11]. A miscarriage or induced abortion provide some protection, confirming immune adaptation theories [12]. Risk factors associated with pre-eclampsia include maternal diabetes, [13,14] chronic hypertension,[13,14] renal disease,[15] thrombophilias,[16,17] and autoimmune disorders [18]. Obstetric factors associated with high risk are multiple pregnancies,[19] previous pre-eclampsia, and molar or hydropic pregnancies [6,20]. Other risk factors are first pregnancy,[6,20] extremes of age,[11] and obesity [21]. A family history of pre-eclampsia may suggest a genetic predisposition [6,20].

Hypertensive disorders remain one of the largest single causes of maternal and foetal mortality and morbidity. Depending upon the region, between 9.1% (Africa, Asia), 16.1% (developed countries), and 25.7% (Latin America) of maternal deaths can be accounted for by these disorders [22]. Up to 18% of foetal deaths are associated with hypertensive disorders [23]. Complications for the women include coagulopathy, renal failure and stroke. For the baby they include preterm delivery and intra uterine growth restriction,[23] which are associated with increased risk of developmental delay and chronic diseases in childhood. In the long term, women and foetuses affected by these disorders may be prone to cardiovascular disease in adult life [24-28]. The vast majority of these complications are related to pre-eclampsia.

Several tests have been purported to predict pre-eclampsia but the underlying evidence often lacks quality and/or precision. Accurate prediction of pre-eclampsia is important, because intensive monitoring and administration of early treatment with e.g. aspirin [29] can be more selectively targeted at high-risk women, making timely intervention easier and possibly more cost-effective. This may prevent some of the mortality and morbidity. However, decision making is currently hampered due to lack of up to date and comprehensive evidence summaries on estimates of risk of developing pre-eclampsia.

Methods/Design

Objective

This research project is undertaken to meet the following objective: to determine, among women in early pregnancy, the accuracy of various tests (history, examination, investigations) for predicting the later development of pre-eclampsia.

Search strategy

Literature will be identified using

• general health and biomedical bibliographies: MEDLINE (PubMED), EMBASE (Ovid)

• specialised electronic databases: The Cochrane Library (DARE, CCTR), MEDION

• contact with experts, including the Cochrane Pregnancy and Childbirth Group

• citation lists of review articles and papers that will be eligible for the reviews in this project.

In a single comprehensive search we will aim to find all primary studies reporting on the accuracy of any test (combinations) used to predict pre-eclampsia. We will combine search terms related to pre-eclampsia with methodological filters for identification of aetiologic and diagnostic test studies (table 1) [30-32]. All databases will be searched from inception. Experienced clinical librarians will perform the searches and their updates. No language restrictions will be applied. A comprehensive master database using Citation Manager 10.0 software will be established incorporating results of all searches.

Inclusion criteria

The study inclusion criteria are:

Population

Any pregnant women in primary, secondary or tertiary care, at any level of risk of developing pre-eclampsia. Stud-

Table 1: Search strategy to identify citations on tests used to predict pre-eclampsia

MEDLINE

1. preeclamp* OR eclamp* OR pre-eclamp* OR (pre AND eclamp*) OR (pregnan* AND hypertens*)
2. ("Eclampsia"[MeSH] OR "Gestosis, EPH"[MeSH] OR ("Hypertension"[MeSH] AND "Pregnancy"[MeSH]))
3. "Sensitivity and Specificity"[MeSH] OR predict* OR diagnose* OR diagnosi* OR diagnost* OR accura*

(1 OR 2) AND 3 *(diagnosis)*

4. (((((("cohort studies"[mh] OR "case-control studies"[MeSH Terms]) OR "risk"[mh]) OR "epidemiologic factors"[MeSH Terms]) OR ("odds"[tw] AND "ratio*"[tw])) OR ("relative"[tw] AND "risk"[tw])) OR ("case"[tw] AND "control*"[tw]))

(1 OR 2) AND 4 *(aetiology)*

EMBASE

1. exp "ECLAMPSIA AND PREECLAMPSIA"/
2. exp PREGNANCY/
3. exp hypertension/
4. 2 and 3
5. 1 or 4
6. (preeclamp$ or eclamp$ or pre-eclamp$ or (pre and eclamp$) or (pregnan$ and hypertens$)).mp.
7. (sensitiv$ or detect$ or accura$ or specific$ or reliab$ or positive or negative or diagnos$).mp. or di.fs.
8. 5 or 6
9. 7 and 8 *(diagnosis)*
10. cohort analysis/
11. exp risk/
12. (odds$ adj ratio$).mp.
13. (relative adj risk).mp.
14. case control study/
15. (case$ adj control$).mp.
16. (causa$ or predispos$).mp.
17. or/10–16
18. 5 or 6
19. 17 and 18 *(aetiology)*

Cochrane Library

Set 1

1. preeclamp* OR eclamp* OR pre-eclamp* OR (pre AND eclamp*) OR (pregnan* AND hypertens*) in All Fields in all procucts

Set 2

2. MeSH descriptor **Eclampsia** explode all trees in MeSH products
3. MeSH descriptor **Hypertension** explode all trees in MeSH products
4. MeSH descriptor **Pregnancy** explode all trees in MeSH products
5. (#2 OR (#3 AND #4))

Set 3

6. MeSH descriptor **Sensitivity and Specificity** explode all trees in MeSH products
7. **Predict* OR diagnose* OR diagnosi* OR diagnost* OR accura*** in All Fields
8. (#6 OR #7)

(1 OR 2) AND 3 *(diagnosis)*

Set 4

1. MeSH descriptor **Cohort Studies** explode all trees in MeSH products
2. MeSH descriptor **Case-Control Studies** explode all trees in MeSH products
3. MeSH descriptor **Risk** explode all trees in MeSH products
4. MeSH descriptor **Epidemiologic Factors** explode all trees in MeSH products
5. **((odds AND ratio) OR (relative AND risk) OR (case AND control))** in All Fields in all products
6. **(#1 OR #2 OR #3 OR #4 OR #5)**

(1 OR 2) AND 4 *(aetiology)*

ies will be included that tested women at risk of developing pre-eclampsia before 25 weeks of gestation. When gestational age at the time of the index test varies the mean gestational age as calculable from the descriptive statistics must be less than 25 weeks. When gestational age is unclear the study will be excluded.

Setting

Any setting including general practice, midwifery, outpatient clinics, or based on national or regional registers.

Study design

Diverse study designs will be included such as prospective cohorts, historic cohorts, and (nested) case-control studies, all of which may be matched or unmatched on different variables. Studies must report results so that a 2 × 2 table cross classifying abnormal and normal test results and the occurrence or non-occurrence of pre-eclampsia can be calculated. We will exclude all cross-sectional studies in which the distribution of a non-stable indicator (often a blood constituent) among women with pre-eclampsia was compared to that of non-pre-eclamptic women.

Predictive tests (index tests)

Tests used for the prediction of pre-eclampsia will be prioritised on the basis of clinical relevance and after consultation with persons knowledgeable of NHS needs (we will consult particularly with the NHS Antenatal Sourcing Subgroup).

Reference standard

Pre-eclampsia, using a variety of definitions. Pre-eclampsia is defined as hypertension (≥ 140/90 mmHg) with proteinuria (total protein of ≥ 300 mg in a 24 hour urine collection, or ≥ 30 mg/dL in a single sample of urine, or ≥ 1+ on a dipstick) developing for the first time after 20 weeks gestation, with or without generalised oedema. For women with chronic hypertension, pre-eclampsia is defined as a sudden worsening of hypertension and/or proteinuria, or other signs and symptoms of pre-eclampsia after 20 weeks gestation. When authors do not provide details of how pre-eclampsia was verified, pre-eclampsia rates as reported will be accepted. At the stage of data-extraction, the extent that pre-eclampsia definition complies with recent consensus will be assessed. All studies that compare a test or strategy with a reference standard according to international standards or variations of the definition, in pregnant women will be included [1-3].

Subgroups

Severe pre-eclampsia is defined as hypertension (systolic blood pressure ≥ 160 mmHg and/or diastolic blood pressure ≥ 110 mmHg) with proteinuria (total protein of ≥ 2.0 gram in a 24 hour urine collection, or ≥ 3+ on a dipstick). A distinction will be made between early onset (< 34 weeks gestation) and late onset (≥ 34 weeks gestation) pre-eclampsia.

Study selection process

The study selection process will consist of three steps. Firstly, titles and/or abstracts of all citations in the master database (that is, irrespective of test type) will be assessed by one reviewer. If a citation is considered potentially relevant, the full-text paper will be retrieved for further consideration. Reviewers will be instructed to include any citation in case of doubt, thus enhancing the sensitivity of the initial selection step. Secondly, for each particular review, a search based on keywords and text words in titles and abstracts in the master database will be performed to find all studies on the test at issue. Another reviewer will scrutinise titles and/or abstracts of studies on the test at issue to ensure independent duplicate selection. Only papers judged irrelevant twice will not be ordered as full-text papers and all other papers will be retrieved. Thirdly, inclusion will be performed independently by two reviewers assessing against the selection criteria detailed above. Disagreements will be resolved either by consensus or by arbitration by a third reviewer when consensus cannot be reached.

Data extraction

Clinical, methodological and statistical data extraction will be conducted independently in duplicate using a pre-

Table 2: Tests for hypertensive disorders in pregnancy

History	Risk factors e.g. nulliparity, new partner, hypertension on oral contraceptives, pre-existing diabetes, renal disease, chronic hypertension, parity
Examination	Blood pressure (systolic and diastolic and mean arterial pressure), peripheral oedema, body mass index, waist circumference, hip:waist ratio
Investigations	
**Biochemical**	Serum uric acid, urinary calcium excretion, urinary albumin-creatinine and calcium-creatinine ratios, microalbuminuria, fibronectin, spot proteinuria, 24-hour urinary protein levels
**Haemodynamic**	Pressor response to various forms of stimuli e.g., supine "rollover", isometric exercise, passive tilting, uterine artery Doppler
**Haematological**	Antithrombin III, platelet count, haemoglobin, haematocrit, fibrinogen
**Other tests**	Thrombomodulin, endothelin-1, plasminogen activator inhibitor, free fatty acids, atrial natriuretic peptide, angiotensin II infusion, platelet angiotensin II binding site density, öhCG, õFP, fasting insulin levels

designed and piloted form that will vary only slightly between different reviews.

The following data will be extracted from each included study: first author; year of publication; setting; number of participating centres; country of investigation; admission criteria for participants; baseline characteristics; inclusion period; study design characteristics; details of the index test measurement; details of the citation standard; numbers of included subjects, proportion dropped out, proportion uninterpretable/indeterminate/intermediate test results; (pre-specified) cut-off values; incidence of pre-eclampsia within the study population; (mean) time of onset and severity of pre-eclampsia; numbers of subjects on which to base 2 × 2 data tables or necessary data for the construction of a 2 × 2 data table; correlation coefficients between tests; financial support of industry.

Again, disagreements between reviewers will be resolved by consensus or by arbitration by a third reviewer when consensus cannot be reached. In case of serial index test measurements during pregnancy a 2 × 2 table will be constructed for each serial measurement. Where multiple publications of the same study will be identified, each publication will be examined to ensure that all relevant data for that particular study are recorded. Only the most complete report will be used for extracting results.

Pairs of data-extraction forms will be checked for discrepancies. After resolution of disagreements, data will be entered into a dedicated SPSS database. Relevant variables will be checked using descriptive statistics to detect implausible values or outliers. Extreme or outlying values will be checked against the original data extraction forms and against the original publications if necessary to further exclude the possibility of data-entry errors.

Quality assessment

Quality items will be included in the data extraction form. The following aspects of methodological and reporting quality of included test accuracy studies will be assessed[33,34].

- study design;
- consecutive recruitment/random sample;
- blinding of test results (both index and reference tests);
- greater than 90% verification of diagnosis;
- incidence of pre-eclampsia less than 4%;
- prospective data collection;
- adequate description of index test;
- adequate reference standard.

Note that items on study design and incidence are not strictly related to internal validity, but help the reader to put the findings in context. Study quality will be assessed independently by two reviewers. Any disagreements will be resolved by consensus or by arbitration by a third reviewer, when consensus cannot be reached.

The following items will be assessed using the three criteria listed under each item:

• *Consecutive recruitment*
Yes – when the test explicitly states consecutive or random sampling;

No – when the description is explicit but words indicating consecutive or random sampling are missing;

Unclear – otherwise.

• *Blinding of test results (index test or reference test)*
Yes – adequate blinding will be judged present when the text explicitly states that the results of each test were interpreted unaware (blind, masked) of the results of the other test or when it may be inferred that they were interpreted before the other test results were available (for example the index test result in prospective cohort studies);

No – inadequate blinding will be judged present if it is clear from the text that neither test result was interpreted under blind conditions;

Unclear – otherwise.

• *Verification of diagnosis*
Yes – adequate verification of diagnosis will be judged present where at least 90% of the women originally subjected to the index test and fulfilling the inclusion criteria were followed up and had verification by the reference standard;

No – inadequate where the follow up percentage is below 90%;

Unclear – where the numbers of women excluded or lost to follow up are not calculable;

• *Incidence of pre-eclampsia < 4%*
Yes – unselected (low risk) patient spectrum where incidence of pre-eclampsia less than or equal to 4%;*

No – selected patient spectrum where incidence of pre-eclampsia is greater than 4%;

Unclear – when the incidence cannot be derived from the article (for example in case control studies).

We intend to dichotomise the item "incidence of pre-eclampsia" using an incidence cut-off value of 4% in cohort studies (and based on the underlying cohort in nested case-control analyses where possible). This seems wise because we found that in cohort studies with more than 30,000 women, which reflect more or less unselected populations, the incidence of pre-eclampsia varies between 1.3 and 3.2% [35-37]

• *Prospective data collection*

Yes – adequate when it is clear that the research protocol for the study has been written before data collection took place (prospective);

No – inadequate when there is retrospective data collection or there is a mixture of prospective and retrospective data collection;;

Unclear – otherwise.

• *Adequate description of index test*

Yes – if the gestational age at the time of testing, type of test (e.g. assay/manufacturer, specified for each test) and cut-off level are reported;

No – if nothing is reported;

Unclear – omission of one or more items.

• *Adequate reference standard*

Yes – if strictly in accordance with current internationally accepted standards of definition of pre-eclampsia;

No – when the definition of pre-eclampsia includes other items such as a rise in systolic or diastolic blood pressure or for example hyperuricaemia or oedema, or when criteria are more loose or stringent;

Unclear – otherwise.

Adequacy of the reference standard for studies that report on severe pre-eclampsia as an outcome will be based on the definition of severe pre-eclampsia.

Methods of statistical analysis

The main focus of each review will be a summary estimate of predictive accuracy as expressed by its sensitivity and specificity. A secondary aim is to identify (clinically relevant) sources of heterogeneity. If the calculation of a summary estimate is judged not meaningful, individual study results will be depicted using forest plots of sensitivity and specificity with their 95% confidence intervals, and using receiver operating characteristics (ROC) plots ("1-specificity" against "sensitivity").

Data exploration and statistical analysis

In each review, we will use forest plots and ROC plots to display the precision by which sensitivity and specificity has been measured in each study, and to illustrate the variation in estimates between studies. Ninety-five percent confidence intervals (95% CIs) will be calculated using the exact binomial method, according to Wilson[38]. Extreme values, outliers, and threshold phenomena (data points on a typical convex ROC curve) will be explored.

We will consider the use of a bivariate meta-regression model to meta-analyse estimates of sensitivity (true positives/(true positives + false negatives) and specificity (true negatives/(true negatives + false positives) [39,40]. Rather than using a single outcome measure per study, like the diagnostic odds ratio in the summary ROC approach, the bivariate model preserves the two-dimensional nature of diagnostic data by directly analysing the logit transformed sensitivity log(sens/(1-sens)) and specificity log(spec/(1-spec)) of each study in a single model. This model estimates and incorporates the correlation that may exist between logit sensitivity and logit specificity within studies due to possible differences in threshold between studies. The bivariate model uses a random effects approach for both sensitivity and specificity, allowing for heterogeneity beyond chance due to clinical or methodological differences between studies. In addition, the model acknowledges the difference in precision by which sensitivity and specificity have been measured in each study. This means that studies with a larger number of patients with the target condition receive more weight in the calculation of the summary estimate of sensitivity, while studies with more patients without the target condition are more influential in the pooling of specificity. A standard correction of adding 0.5 to all four cells of the 2×2 table will be applied when either sensitivity or specificity is 100%. The model produces the following results: a random effect estimate of the mean sensitivity and specificity with corresponding 95% confidence intervals, the amount of between-study variation for sensitivity and specificity separately, and the strength and shape of the correlation between sensitivity and specificity. All the results will be transformed back (anti-logit) to the original scale, and plotted in ROC space. Where possible, covariates will be added to the model to test explicitly whether either sensitivity, or specificity, or both are different in studies with and without the characteristic. When possible the analysis aims to estimate valid measures of predictive accuracy taking into account confounding by any methodological

flaws. In a first instance, attempts will be always made to quantify the extent to which the accuracy measures varied by clinical subgroups, such as early versus late testing. STATA/SE 9.0 (StataCorp, Texas, USA) will be used for calculations except to fit the various bivariate models for which the Proc Mixed procedure in SAS version 9.1 for Windows (SAS Institute Inc, Cary, NC, USA) will be used.

Discussion

The methodology of systematic reviews of test accuracy is difficult and still developing. In contrast to the field of randomised trials, didactic guidelines on conducting primary diagnostic studies and systematic reviews of diagnostic studies have only recently been developed [41-43]. Also more recently, a quality tool for diagnostic studies has been developed [44]. Older studies tend to report less accurately than more recent studies, which does not necessarily prove that older studies executed less rigorously than more recent studies. Therefore, incorporating methodological and reporting quality of primary studies in analyses of systematic reviews may be difficult. However, shortcomings in design and conduct can affect estimates of diagnostic accuracy, but the magnitude of the effect may vary from one situation to another [45].

This project is part of a more comprehensive research project concerning prediction and prevention of pre-eclampsia. The results of the accuracy reviews will be integrated with results of effectiveness reviews of preventive interventions to assess cost-effectiveness of strategies (test-intervention combinations) for prediction and prevention of pre-eclampsia. Results will probably be available in 2007.

Competing interests

The author(s) declare that they have not competing interests.

Authors' contributions

JC worked out the protocol, developed searches and data management, will participate in selection, inclusion, quality assessment and data extraction of papers for several reviews, will perform statistical analyses and wrote the first draft of the manuscript. JP and BW helped working out the protocol and will participate in performing several test accuracy reviews. KK is the general project supervisor, conceived the original integrated research project and advised on the content of the project. CM will participate in performing several test accuracy reviews. GR is the project supervisor for test accuracy reviews and helped working out the protocol and will participate in performing several test accuracy reviews and writing drafts. All authors read and approved the final manuscript.

Acknowledgements

We thank clinical librarians Marjan Loep (†) and Faridi van Etten for their advise and help with literature searches. We thank Mariska Leeflang, Hans Reitsma and Koos Zwinderman from the AMC Department of Clinical Epidemiology and Biostatistics for their advise and help with statistical analyses.

This study is financed by the NHS Health Technology Assessment UK, project code 01/64.

References

1. **Report of the National High Blood Pressure Education Program Working Group on High Blood Pressure in Pregnancy.** *Am J Obstet Gynecol* 2000, **183:**S1-S22.
2. Brown MA, Hague WM, Higgins J, Lowe S, McCowan L, Oats J, Peek MJ, Rowan JA, Walters BN: **The detection, investigation and management of hypertension in pregnancy: full consensus statement.** *Aust N Z J Obstet Gynaecol* 2000, **40:**139-155.
3. Brown MA, Lindheimer MD, de Swiet M, Van Assche A, Moutquin JM: **The classification and diagnosis of the hypertensive disorders of pregnancy: statement from the International Society for the Study of Hypertension in Pregnancy (ISSHP).** *Hypertens Pregnancy* 2001, **20:**IX-XIV.
4. Levine RJ, Ewell MG, Hauth JC, Curet LB, Catalano PM, Morris CD, Choudhary G, Sibai BM: **Should the definition of preeclampsia include a rise in diastolic blood pressure of >/=15 mm Hg to a level <90 mm Hg in association with proteinuria?** *Am J Obstet Gynecol* 2000, **183:**787-792.
5. North RA, Taylor RS, Schellenberg JC: **Evaluation of a definition of pre-eclampsia.** *Br J Obstet Gynaecol* 1999, **106:**767-773.
6. Sibai B, Dekker G, Kupferminc M: **Pre-eclampsia.** *Lancet* 2005, **365:**785-799.
7. Roberts JM, Redman CW: **Pre-eclampsia: more than pregnancy-induced hypertension.** *Lancet* 1993, **341:**1447-1451.
8. Walker JJ: **Pre-eclampsia.** *Lancet* 2000, **356:**1260-1265.
9. Khong TY, De Wolf F, Robertson WB, Brosens I: **Inadequate maternal vascular response to placentation in pregnancies complicated by pre-eclampsia and by small-for-gestational age infants.** *Br J Obstet Gynaecol* 1986, **93:**1049-1059.
10. Einarsson JI, Sangi-Haghpeykar H, Gardner MO: **Sperm exposure and development of preeclampsia.** *Am J Obstet Gynecol* 2003, **188:**1241-1243.
11. Dekker G, Robillard PY: **The birth interval hypothesis - Does it really indicate the end of the primipaternity hypothesis.** *Journal of Reproductive Immunology* 2003, **59:**245-251.
12. Strickland DM, Guzick DS, Cox K, Gant NF, Rosenfeld CR: **The relationship between abortion in the first pregnancy and development of pregnancy-induced hypertension in the subsequent pregnancy.** *Am J Obstet Gynecol* 1986, **154:**146-148.
13. Caritis S, Sibai B, Hauth J, Lindheimer M, Vandorsten P, Klebanoff M, Thom E, Landon M, Paul R, Miodovnik M, Meis P, Thurnau G, Dombrowski M, McNellis D, Roberts J: **Predictors of pre-eclampsia in women at high risk. National Institute of Child Health and Human Development Network of Maternal-Fetal Medicine Units.** *Am J Obstet Gynecol* 1998, **179:**946-951.
14. Conde-Agudelo A, Belizan JM: **Risk factors for pre-eclampsia in a large cohort of Latin American and Caribbean women.** *BJOG* 2000, **107:**75-83.
15. Cunningham FG, Cox SM, Harstad TW, Mason RA, Pritchard JA: **Chronic renal disease and pregnancy outcome.** *Am J Obstet Gynecol* 1990, **163:**453-459.
16. Alfirevic Z, Roberts D, Martlew V: **How strong is the association between maternal thrombophilia and adverse pregnancy outcome? A systematic review.** *Eur J Obstet Gynecol Reprod Biol* 2002, **101:**6-14.
17. Robertson L, Wu O, Langhorne P, Twaddle S, Clark P, Lowe GD, Walker ID, Greaves M, Brenkel I, Regan L, Greer IA: **Thrombophilia in pregnancy: a systematic review.** *Br J Haematol* 2006, **132:**171-196.
18. Gordon C: **Pregnancy and autoimmune diseases.** *Best Pract Res Clin Rheumatol* 2004, **18:**359-379.
19. Sibai BM, Hauth J, Caritis S, Lindheimer MD, MacPherson C, Klebanoff M, VanDorsten JP, Landon M, Miodovnik M, Paul R, Meis P, Thurnau G, Dombrowski M, Roberts J, McNellis D: **Hypertensive disorders**

in twin versus singleton gestations. National Institute of Child Health and Human Development Network of Maternal-Fetal Medicine Units. *Am J Obstet Gynecol* 2000, **182:**938-942.
20. Dekker G, Sibai B: **Primary, secondary, and tertiary prevention of pre-eclampsia.** *Lancet* 2001, **357:**209-215.
21. O'Brien TE, Ray JG, Chan WS: **Maternal body mass index and the risk of preeclampsia: a systematic overview.** *Epidemiology* 2003, **14:**368-374.
22. Khan KS, Wojdyla D, Say L, Gulmezoglu AM, Van Look PF: **WHO analysis of causes of maternal death: a systematic review.** *Lancet* 2006, **367:**1066-1074.
23. Montan S, Sjoberg NO, Svenningsen N: **Hypertension in pregnancy - Fetal and infant outcome. A cohort study.** *Clinical & Experimental Hypertension - Part B, Hypertens Pregnancy* 1987, **6:**337-348.
24. Haukkamaa L, Salminen M, Laivuori H, Leinonen H, Hiilesmaa V, Kaaja R: **Risk for subsequent coronary artery disease after preeclampsia.** *Am J Cardiol* 2004, **93:**805-808.
25. Ramsay JE, Stewart F, Greer IA, Sattar N: **Microvascular dysfunction: a link between pre-eclampsia and maternal coronary heart disease.** *BJOG* 2003, **110:**1029-1031.
26. Wilson BJ, Watson MS, Prescott GJ, Sunderland S, Campbell DM, Hannaford P, Smith WC: **Hypertensive diseases of pregnancy and risk of hypertension and stroke in later life: results from cohort study.** *BMJ* 2003, **326:**845.
27. Hack M, Flannery DJ, Schluchter M, Cartar L, Borawski E, Klein N: **Outcomes in young adulthood for very-low-birth-weight infants.** *N Engl J Med* 2002, **346:**149-157.
28. Barker DJ: **The developmental origins of chronic adult disease.** *Acta Paediatr Suppl* 2004, **93:**26-33.
29. Duley L, Henderson-Smart DJ, Knight M, King JF: **Antiplatelet agents for preventing pre-eclampsia and its complications.** *Cochrane Database Syst Rev* 2004:CD004659.
30. *http://info med yale edu/library/reference/publications/pubmed/* 2003 [http://info.med.yale.edu/library/reference/publications/pubmed/].
31. Bachmann LM, Coray R, Estermann P, Ter Riet G: **Identifying diagnostic studies in MEDLINE: reducing the number needed to read.** *J Am Med Inform Assoc* 2002, **9:**653-658.
32. Haynes RB, Wilczynski N, McKibbon KA, Walker CJ, Sinclair JC: **Developing optimal search strategies for detecting clinically sound studies in MEDLINE.** *J Am Med Inform Assoc* 1994, **1:**447-458.
33. Lijmer JG, Mol BW, Heisterkamp S, Bonsel GJ, Prins MH, van der Meulen JH, Bossuyt PM: **Empirical evidence of design-related bias in studies of diagnostic tests.** *JAMA* 1999, **282:**1061-1066.
34. Whiting P, Rutjes AW, Reitsma JB, Bossuyt PM, Kleijnen J: **The development of QUADAS: a tool for the quality assessment of studies of diagnostic accuracy included in systematic reviews.** *BMC Med Res Methodol* 2003, **3:**25.
35. Waller DK, Lustig LS, Cunningham GC, Feuchtbaum LB, Hook EB: **The association between maternal serum alpha-fetoprotein and preterm birth, small for gestational age infants, preeclampsia, and placental complications.** *Obstet Gynecol* 1996, **88:**816-822.
36. Yaron Y, Cherry M, Kramer RL, O'Brien JE, Hallak M, Johnson MP, Evans MI: **Second-trimester maternal serum marker screening: maternal serum alpha-fetoprotein, beta-human chorionic gonadotropin, estriol, and their various combinations as predictors of pregnancy outcome.** *Am J Obstet Gynecol* 1999, **181:**968-974.
37. Yu CK, Smith GC, Papageorghiou AT, Cacho AM, Nicolaides KH: **An integrated model for the prediction of preeclampsia using maternal factors and uterine artery Doppler velocimetry in unselected low-risk women.** *Am J Obstet Gynecol* 2005, **193:**429-436.
38. Newcombe RG: **Two-sided confidence intervals for the single proportion: comparison of seven methods.** *Stat Med* 1998, **17:**857-872.
39. Van Houwelingen HC, Zwinderman KH, Stijnen T: **A bivariate approach to meta-analysis.** *Stat Med* 1993, **12:**2273-2284.
40. Van Houwelingen HC, Arends LR, Stijnen T: **Advanced methods in meta-analysis: multivariate approach and meta-regression.** *Stat Med* 2002, **21:**589-624.
41. Bossuyt PM, Reitsma JB, Bruns DE, Gatsonis CA, Glasziou PP, Irwig LM, Moher D, Rennie D, de Vet HC, Lijmer JG: **The STARD statement for reporting studies of diagnostic accuracy: explanation and elaboration.** *Clin Chem* 2003, **49:**7-18.
42. Bossuyt PM, Reitsma JB, Bruns DE, Gatsonis CA, Glasziou PP, Irwig LM, Lijmer JG, Moher D, Rennie D, de Vet HC: **Towards complete and accurate reporting of studies of diagnostic accuracy: The STARD Initiative.** *Ann Intern Med* 2003, **138:**40-44.
43. Deville WL, Buntinx F, Bouter LM, Montori VM, de Vet HC, van der Windt DA, Bezemer PD: **Conducting systematic reviews of diagnostic studies: didactic guidelines.** *BMC Med Res Methodol* 2002, **2:**9.
44. Whiting P, Rutjes AW, Dinnes J, Reitsma J, Bossuyt PM, Kleijnen J: **Development and validation of methods for assessing the quality of diagnostic accuracy studies.** *Health Technol Assess* 2004, **8:**iii, 1-iii234.
45. Rutjes AW, Reitsma JB, Di Nisio M, Smidt N, van Rijn JC, Bossuyt PM: **Evidence of bias and variation in diagnostic accuracy studies.** *CMAJ* 2006, **174:**469-476.

Pre-publication history

The pre-publication history for this paper can be accessed here:

http://www.biomedcentral.com/1471-2393/6/29/prepub

Publish with **BioMed Central** and every scientist can read your work free of charge

"BioMed Central will be the most significant development for disseminating the results of biomedical research in our lifetime."

Sir Paul Nurse, Cancer Research UK

Your research papers will be:

- available free of charge to the entire biomedical community
- peer reviewed and published immediately upon acceptance
- cited in PubMed and archived on PubMed Central
- yours — you keep the copyright

Submit your manuscript here:
http://www.biomedcentral.com/info/publishing_adv.asp

BioMedcentral
