# Supplementary material for: Serum screening with Down's syndrome markers to predict pre-eclampsia and small for gestational age: Systematic review and meta-analysis
Source: BMC Pregnancy Childbirth. 2008 Aug 4;8:33. doi: 10.1186/1471-2393-8-33 (PMC2533288; doi:10.1186/1471-2393-8-33)
Supplement: Additional file 3 — "Search strategies for biochemical markers used in Down's serum screening to predict preeclampsia/small for gestational age." Electronic search strategies for systematic reviews. [file 1471-2393-8-33-S3.doc]

**Supplemental file S3 – Search strategies for biochemical markers used in Down’s serum screening to predict preeclampsia/ small for gestational age**

**Preeclampsia**

*MEDLINE*

1. preeclamp* OR eclamp* OR pre-eclamp* OR (pre AND eclamp*) OR (pregnan* AND hypertens*)
2. ("Eclampsia"[MeSH] OR "Gestosis, EPH"[MeSH] OR ("Hypertension"[MeSH] AND "Pregnancy"[MeSH]))
3. "Sensitivity and Specificity"[MeSH] OR predict* OR diagnose* OR diagnosi* OR diagnost* OR accura*
4. (1 OR 2) AND 3 *diagnosis*
5. (((((("cohort studies"[mh] OR "case-control studies"[MeSH Terms]) OR "risk"[mh]) OR "epidemiologic factors"[MeSH Terms]) OR ("odds"[tw] AND "ratio*"[tw])) OR ("relative"[tw] AND "risk"[tw])) OR ("case"[tw] AND "control*"[tw]))
6. (1 OR 2) AND 5 a*etiology*

*EMBASE*

1. exp "ECLAMPSIA AND PREECLAMPSIA"/
2. exp PREGNANCY/
3. exp hypertension/
4. 2 and 3
5. 1 or 4
6. (preeclamp$ or eclamp$ or pre-eclamp$ or (pre and eclamp$) or (pregnan$ and hypertens$)).mp.
7. (sensitiv$ or detect$ or accura$ or specific$ or reliab$ or positive or negative or diagnos$).mp. or di.fs.
8. 5 or 6
9. 7 and 8
10. cohort analysis/
11. exp risk/
12. (odds$ adj ratio$).mp.
13. (relative adj risk).mp.
14. case control study/
15. (case$ adj control$).mp.
16. (causa$ or predispos$).mp.
17. or/10-16
18. 5 or 6
19. 17 and 18

*COCHRANE LIBRARY*

1. (preeclamp*) or (eclamp*) or (pre-eclamp*) or (pre AND eclamp*) or pregnan* AND hypertens*) in All Fields in all products
2. MeSH descriptor **Eclampsia** explode all trees in MeSH products
3. MeSH descriptor **Hypertension** explode all trees in MeSH products
4. MeSH descriptor **Pregnancy** explode all trees in MeSH products
5. (#2 OR #3 OR #4)
6. MeSH descriptor **Sensitivity and Specificity** explode all trees in MeSH products
7. **(predict* OR diagnose* OR diagnosi* OR diagnost* OR accura*)** in All Fields in all products
8. (#6 OR #7)
9. (( #1 OR #5) AND #8)
10. MeSH descriptor **Cohort Studies** explode all trees in MeSH products
11. MeSH descriptor **Case-Control** Studies explode all trees in MeSH products
12. MeSH descriptor **Risk** explode all trees in MeSH products
13. MeSH descriptor **Epidemiologic Factors** explode all trees in MeSH products
14. (odds AND ratio) OR (relative AND risk) OR (case AND control) in All Fields in all products
15. (#10 OR #11 OR #12 OR #13 OR #14)
16. (( #1 OR #5) AND #15)

**Fetal growth restriction**

*MEDLINE*

1. (("Small-for-Gestational Age") OR (Small-for-Gestational Age) OR (lbw) OR (small for gestational age) OR (sgr) OR (small for date*) OR (small for gestation*) OR (fgr) OR (iugr) OR (intrauterine growth retard*) OR (intrauterine growth restrict*) OR (fetal growth retard*) OR (fetal growth restrict*) OR (growth restrict*) OR (growth retard*) OR ("Placental Insufficiency"[MeSH]) OR ("Fetal Growth Retardation"[MeSH]) OR ("Infant, Low Birth Weight"[MeSH])) OR (low birth weight)
2. ("Pregnant Women"[MeSH] OR "Pregnancy"[MeSH] OR "Pregnancy Outcome"[MeSH]) OR (pregnan*)
3. Sensitivity and Specificity[MeSH] OR predict* OR diagnose* OR diagnosi* OR diagnost* OR accura*
4. 1 AND 2 AND 3
5. ((((("cohort studies"[mh] OR "case-control studies"[MeSH Terms]) OR "risk"[mh]) OR "epidemiologic factors"[MeSH Terms]) OR ("odds"[tw] AND "ratio*"[tw])) OR ("relative"[tw] AND "risk"[tw])) OR ("case"[tw] AND "control*"[tw])
6. 1 AND 2 AND 5

*EMBASE*

1. exp Fetus Growth/
2. low birth weight.mp. or exp Low Birth Weight/
3. exp Intrauterine Growth Retardation/
4. Intrauterine Growth Retard$.mp.
5. Growth Retard$.mp.
6. Fetal Growth Retard$.mp.
7. intrauterine growth restrict$.mp.
8. fetal growth restrict$.mp.
9. growth restrict$.mp.
10. exp Small for Date Infant/
11. Small for gestational age.mp.
12. Small for date$.mp.
13. Small for gestation$.mp.
14. fgr.mp.
15. iugr.mp.
16. sga.mp.
17. or/1-16
18. exp pregnancy/
19. exp Pregnant Woman/
20. pregnancy outcome.mp.
21. pregnan$.mp.
22. pregnant wom$.mp.
23. exp Placenta Insufficiency/
24. or/18-23
25. (sensitiv$ or detect$ or accura$ or specific$ or reliab$ or positive or negative or diagnos$).mp. or di.fs.
26. 17 and 24 and 25
27. cohort analysis/
28. exp risk/
29. (odds$ adj ratio$).mp.
30. (relative adj risk).mp.
31. case control study/
32. (case$ adj control$).mp.
33. (causa$ or predispos$).mp.
34. or/27-33
35. 17 and 24 and 34

*COCHRANE LIBRARY*

1. **small for gestational age** in All Fields in all products
2. **sga** in All Fields in all products
3. **small for date** in All Fields in all products
4. **fgr** in All Fields in all products
5. **lbw** in All Fields in all products
6. **iugr** in All Fields in all products
7. **intrauterine growth retard*** in All Fields in all products
8. **fetal growth retardation** in All Fields in all products
9. **fetal growth retard*** in All Fields in all products
10. **growth restrict*** in All Fields in all products
11. **growth retard*** in All Fields in all products
12. **low birth weight** in All Fields in all products
13. MeSH descriptor **Placental Insufficiency** explode all trees in MeSH products
14. **placental insufficiency** in All Fields in all products
15. (#1 OR #2 OR #3 OR #4 OR #5 OR #6 OR #7 OR #8 OR #9 OR #10 OR #11 OR #12 OR #13 OR #14)
16. **pregnancy** in All Fields in all products
17. MeSH descriptor **Pregnant Women** explode all trees in MeSH products
18. MeSH descriptor **Pregnancy** explode all trees in MeSH products
19. MeSH descriptor **Pregnancy Outcome** explode all trees in MeSH products
20. (#16 OR #17 OR #18 OR #19)
21. MeSH descriptor **Sensitivity and Specificity** explode all trees in MeSH products
22. **predict* OR diagnose* OR diagnosi* OR diagnost*** in All Fields in all products
23. (#21 OR #22)
24. (#15 AND #20 AND #23)
25. MeSH descriptor **Cohort Studies** explode all trees in MeSH products
26. MeSH descriptor **Case-Control** Studies explode all trees in MeSH products
27. MeSH descriptor **Risk** explode all trees in MeSH products
28. MeSH descriptor **Epidemiologic Factors** explode all trees in MeSH products
29. (odds AND ratio) OR (relative AND risk) OR (case AND control) in All Fields in all products
30. (#25 OR #26 OR #27 OR #28 OR #29)
31. (#15 AND #20 AND #30)

**Down’s serum screening**

*Reference* *Manager 11.0*

**Alpha feto-protein (AFP)**

KEYWORDS: {alpha-Fetoproteins} OR {Alpha Fetoprotein*} OR {Fetal proteins}

OR TITLE: {alpha-Fetoproteins} OR {Alpha Fetoprotein\*} OR {Fetal proteins}

OR ABSTRACT: {alpha-Fetoproteins} OR {Alpha Fetoprotein\*} OR {Fetal proteins} OR {fetal protein}

**Human chorionic gondaotrophin (HCG)**

KEYWORDS: {*chorion*} OR {*gonadotrophin*} OR {gonadotropin}

OR TITLE: {chorion} OR {gonadotrophin} OR {hcg} OR {gonadotropin} OR {chorionic} OR {chorio}

OR ABSTRACT: {chorion} OR {gonadotrophin} OR {gonadotropin} OR {chorionic} OR {chorio}

**Unconjugated Estriol**

KEYWORDS: {*estradiol*} OR {*estriol*} OR {*estrogen*}

OR TITLE: {Estradiol} OR {Estriol} OR {Estrogen} OR {estrogens}

OR ABSTRACT: {Estradiol} OR {Estriol} OR {Estrogen} OR {estrogens}

**Inhibin and Papp-a**

KEYWORDS: {Pregnancy Associated Plasma Protein*} OR {inhibin*}

OR TITLE: {Pregnancy Associated Plasma Protein\*} OR {inhibin\*} OR {PAPP-A}

OR ABSTRACT: {Pregnancy Associated Plasma Protein\*} OR {inhibin\*} OR {PAPP-A}

**Plasma Proteins**

KEYWORDS: {*Plasma Protein*} OR {inhibin*}

OR TITLE: {\\\*Plasma Protein\*} OR {inhibin\*} OR {PAPP-A}

OR ABSTRACT: {\\\*Plasma Protein\*} OR {inhibin\*} OR {PAPP-A

Note: The search strategy consisted of MeSH or keyword terms related to the disease and population (preeclampsia/ FGR/ pregnant women) combined with methodological filters for identification of studies on diagnostic tests and aetiology^1-3^ to cover the greater topics ‘prediction of preeclampsia’ and ‘prediction of fetal growth restriction’. For this review a search on serum screening markers used in Downs’ screening was performed in the established Reference Manager 11.0 databases.
