# Supplementary material for: Serum screening with Down's syndrome markers to predict pre-eclampsia and small for gestational age: Systematic review and meta-analysis
Source: BMC Pregnancy Childbirth. 2008 Aug 4;8:33. doi: 10.1186/1471-2393-8-33 (PMC2533288; doi:10.1186/1471-2393-8-33)
Supplement: Additional file 4 — "Guide to QUADAS for Down's syndrome markers to predict pre-eclampsia/small for gestational age." Guide to quality assessment of included papers in review using QUADAS tool. [file 1471-2393-8-33-S4.doc]

| Feature | Quadas Number | Applicability and criteria fulfilled when |
| --- | --- | --- |
| **Population spectrum** | 1 | Refers to severity of underlying target condition, demographic features and presence of differential diagnoses and/or co-morbidity. For study to be classified as adequate: Appropriate spectrum – pregnant women, either unselected or selected (high or low risk) in any health care setting. Ideally there was prospective, consecutive recruitment. |
| Selection Criteria | 2 | Refers to inclusion/exclusion criteria. For an unselected population this would not be applicable. For a selected population high risk conditions must be explicitly documented. If the inclusion criteria for the categories were not explicitly described then the category was unclear. |
| **Appropriate Reference standard** | 3 | Preeclampsia: SBP ≥ 140 mmHg and/or DBP ≥ 90 mmHg with proteinuria ≥ 0.3 g/ 24 hours or + dipstick (= 30 mg/dl in single urine sample). Superimposed preeclampsia: proteinuria ≥ 0.3 g/ 24 hours or + dipstick after 20 weeks of gestation in chronically hypertensive patients. Severe preeclampsia: SBP ≥ 160mmHg and/or DBP ≥110mmHg with proteinuria ≥ 2.0g in a 24 hr collection, or ≥3+ on a dipstick.  SGA: birth weight < 10^th^ centile adjusted for gestational age and based on local population values and absolute birth weight threshold < 2500g. Severe SGA: birth weight < 5^th^ or < 3^rd^ centile or < 1750g. Neonatal ponderal index < 10^th^ centile, skin fold thickness, and mid-arm circumference/head circumference were also assessed. |
| **Time period between tests** | 4 | Time period needs to be short enough to ensure that target condition does not change. For this review this was always graded as N/A. |
| Verification Bias | 5 | If >90% of patients or a random selection of patients received verification with reference standard then answer was yes, even if the reference standard was not the same for all patients. If the number was <90% or a non-random selection then the answer was no. Unclear was utilised when the percentage could not be calculated or no information was given. |
| **Number of reference standards used** | 6 | This is N/A to this review: no invasive reference test. |
| **Independent reference standard** | 7 | The results of the index test are not incorporated in the definition of pre-eclampsia/fetal growth restriction. For this review the answer will always be yes. |
| **Adequate description of index test** | 8 | To be graded as adequate the description must include: cut-off used, assay used and manufacturer of assay/machine used. |
| **Adequate description of reference standard** | 9 | Blood pressure: instrument, position of patient, Korotkoff sound for diastolic blood pressure.  Proteinuria: 24 hour collection or dipstick with and cut off.  Birth weight: timing of measurement, scales used, whether baby clothed or not.  Neonatal ponderal index: description of birth weight and length measurement as above.  Skin fold thickness: description of site of measurement, instrument used and timing of measurement.  Mid-arm circumference/ head circumference: see skin fold thickness.  If this information was not provided this was classified as unclear. |

| Blinding of index test | 10 | For this review this answer will always be yes, as the reference standards can only be performed after delivery. In the case of retrospective analysis of blood samples this will also be yes as fully automated. |
| --- | --- | --- |
| **Blinding of reference standard** | 11 | To confirm that blinding was present a statement in the text to the effect of “clinicians were blinded/unaware of the results of the --- test”. If there was a statement to the contrary the answer was no. If no statement existed the answer was unclear. If test were entirely objective (or an independent laboratory was used) then this was N/A. |
| **Availability of clinical data** | 12 | Clinical data refers to any information relating to the patient obtained by direct observation (e.g. age, sex, symptoms, BMI). If clinical data will be available when the test is interpreted in practice then this should be available when the test is evaluated. In this review the test was fully automated and thus is N/A. |
| Intermediate results | 13 | If uninterpretable, failed or intermediate results are documented or no such events occurred then the answer is yes. If it was apparent that such results have occurred but are not reported then the answer was no. If not clear whether all results were reported then answer was unclear. |
| **Withdrawals from study** | 14 | If clear what happened to all patients within the study e.g. flow diagram then answer was yes. If some did not receive both index and reference standard then answer was no. |
| Intervention | A | If after receiving the index test patients received any medical or surgical intervention then the answer was yes, and the type of intervention recorded. If a statement existed that no intervention was given the answer was no. If no statement existed and no interventions were given then the answer was unclear. |
