# Supplementary material for: Serum screening with Down's syndrome markers to predict pre-eclampsia and small for gestational age: Systematic review and meta-analysis
Source: BMC Pregnancy Childbirth. 2008 Aug 4;8:33. doi: 10.1186/1471-2393-8-33 (PMC2533288; doi:10.1186/1471-2393-8-33)
Supplement: Additional file 5 — "Study characteristics of studies of included studies for maternal serum biochemical (Down syndrome) screening to predict pre-eclampsia and small for gestational age." [file 1471-2393-8-33-S5.doc]

| **First Author (year)** | | Population **Age**  **(country/study design)** | | **No of women analysed** | | **Gestational age at test (weeks)** | | **Incidence of PET (%)** | | **Reference standard PET** | | **Incidence of SGA (%)** | | **Reference standard SGA** | | Details of Index test | |
| --- | --- | --- | --- | --- | --- | --- | --- | --- | --- | --- | --- | --- | --- | --- | --- | --- | --- |
| Akinbiyi (76)  (1996) | | INC: singletons, 12% primips, EXC: structural and chromosomal anomalies  18-47 years  (UK) (case control, matched, index test) | | 300 | | 16-18 | | NA | | NA | | 7.33 | | BW <2500g | | AFP,RIA  >2.0 MoM | |
| Aquilina (8)  (2000) | | EXC: multiple pregnancy, IDDM, hypertension < 20 weeks, chromosomal or structural anomalies  Mean age not reported  (UK) (cohort) | | 640 | | 15-19 | | 5.5 | | DBP≥90mmHg 2x 24 hrs or DBP≥110 mmHg, proteinuria >300mg/24hrs or ≥2+ 2x 4hrs apart | | NA | | NA | | Free ßHCG ELISA >2.3MoM | |
| Aquilina (40)  (2001) | | EXC: multiple pregnancy, IDDM, hypertension < 20 weeks, chromosomal or structural anomalies  Mean age not reported  (UK) (cohort) | | 640 | | 15-19 | | 5.47 | | DBP≥90mmHg 2x 24 hrs or DBP≥110 mmHg, proteinuria >300mg/24hrs or ≥2+ 2x 4 hrs apart  (Preterm PET) | | NA | | NA | | Inhibin A, ELISA (Serotec UK),  >0.5->2.0 MoM | |
| Ashour (52)  (1997) | | INC: singletons EXC: foetal/chromosomal anomalies, IDDM, chromic hypertension  Mean age 28.1+/-5.3 years  (USA) (cohort) | | 6138 | | 15-22 | | 3.2 | | SBP≥140mmHg or DBP≥90mmHg 2x 6hrs apart, proteinuria>300mg/24 hrs or ≥1+ 2x 6hrs apart | | NA | | NA | | ßHCG (Imx Abbott) >2.0 MoM | |
| Audibert (64)  (2005) | | INC: double test and uterine artery Doppler 18-26 weeks, singleton, primips 48.5% EXC: structural and chromosomal abnormalities, multiple pregnancies, increased NT, delivery<24 weeks, 8 lost to follow up.  Mean age 30.9+/-4.5 years (France) (cohort) | | 2615 | | 14-18 | | 1.95 | | SBP≥140, DBP≥90 2x, proteinuria >0.3g/24hrs or ≥2+ . | | 8.70 | | BW<10^th^ centile (local) | | Method not reported  AFP>1.5MoM  HCG>1.5 MoM, >2.0MoM | |
|  | |  | |  | |  | |  | |  | |  | |  | |  | |
| Ay (68)  (2005) | | EX: multiple pregnancy, hypertension < 26 weeks, diabetes, chromosomal/ structural abnormality, previous PET  Mean age 28.9 +/-5.1 years(Turkey) (Cohort, prospective) | | 178 | | 16-18 | | 7.87 | | BP>140/90 mmHg after 20 weeks; proteinuria>0.3g/24hrs | | NA | | NA | | AFP, chemiluminescent immunoassay  >1.28 MoM (ROC)  HCG, chemiluminescent immunoassay  >1.75 MoM (ROC)  Inhibin A, immunoassay (Serotec),  >2.79 MoM (ROC) | |
| Benn (77) (1996) | | INC: singletons EXC: structural and chromosomal anomalies, IDDM  Age not reported  (USA) (case control, matched test) | | 1079 | | 15-21.9 | | NA | | NA | | 3.06 | | BW<10th centile | | HCG, Method not reported  >3 MoM | |
| Bernstein (78)  (1992) | | INC: singletons EXC: structural and chromosomal abnormalities  26.3+/-4.3 years  (USA) (cohort, prospective) | | 234 | | 17-21 | | NA | | NA | | 10.7 | | SGA (no threshold) | | AFP, Method not reported  >2.0 MoM | |
|  | |  | |  | |  | |  | |  | |  | |  | |  | |
| Bewley (79)  (1992) | | | INC: singletons  Age not reported  (UK)   | (Cohort, prospective) | | --- | | | --- | --- | | | 172 | | 16-24 | | NA | | NA | | 14.5 | | BW<10th centile (local) | | AFP and HCG,RIA  >90th centile  PAPP-A, RIA >90th and <10th centile | |
| Bloxam (80)  (1994) | | | INC: singletons EXC: structural and chromosomal abnormalities  Age not reported  (UK)(cohort) | | --- | |  | | | 147 | | 16-18 | | NA | | NA | | 14.3 | | BW≤10th centile | | AFP, Method not reported  >1.7MoM | |
| Brajenovic-Milic (65)  (2004) | | INC: singletons, primips 58.5% EXC: IDDM, structural and chromosomal anomalies, false positive NTD screen (MSAFP≥2.o MoM), screen positive downs test ≥1:250, smokers  Mean age 27.9+/-4.3 years  (Croatia) (cohort) | | 1507 | | 15-20 | | 1.66 | | BP>140/90 after 20 weeks with proteinuria +/- oedema | | 4.45 | | BW<10th centile | | Free ßHCG  Method not reported  ≥2.0 MoM | |
| Brazerol (81)  (1994) | | INC: singletons EXC: structural and chromosomal abnormalities  Mean 23.8 years  (USA)(cohort, prospective) | | 774 | | 15-20 | | NA | | NA | | 4.52 | | IUGR (no threshold) | | AFP, Method not reported  ≥2.0MoM | |
| Bremme (82)  (1988) | | INC: singletons EXC: structural and chromosomal abnormalities  Age not reported  (Sweden)(case control) | | 222 | | 16-17 | | NA | | NA | | 7.66 | | SGA (no threshold) | | AFP, RIA (Behringwerke)  >83.3µg/l | |
| Brock (83)  (1980) | | INC: singletons  Age not reported  (UK)(Case control, retrospective, outcome) | | 226 | | 15-22 | | NA | | NA | | 50.0 | | BW<2500g | | AFP, RIA  ≥1.0,1.5,2.0,2.5,3.0 MoM | |
| Buckland (84)  (1984) | | INC: singletons  Age not reported  (UK)(Case control, retrospective, outcome) | | 325 | | 16-20 | | NA | | NA | | 62.5 | | BW<10th centile (sex, parity, local) | | AFP,RIA  ≥2.0 MoM | |
| Burton (85)  (1988) | | INC: screening programme EXC: oligohydramnios  Age not reported  (USA)(Case control, nested cohort) | | 15512 | | 16-18 | | NA | | NA | | 7.33 | | BW<2500g | | AFP, RIA/EIA  (Amersham/Abbott)  >2.5, <0.5 MoM | |
| Capeless (61)  (1992) | | INC: screening programme  Age not reported  (USA) (cohort, prospective) | | 358 | | 16-20 | | 2.0 | | Not reported | | 3.91 | | BW<10th centile | | AFP, Method not reported  >2.0 MoM | |
| Chapman (86)  (1997) | | INC: maternal age ≥30, amniocentesis EXC: structural and chromosomal anomalies  (USA) (cohort, retrospective) | | 1135 | | 15-20 | | NA | | NA | | 3.44 | | BW<10th centile (local) | | Triple test, RIA DS>1:190 | |
| Chard (87)  (1986) | | INC: singletons EXC: delivery < 28 weeks  Age not reported  (UK) (cohort, prospective) | | 476 | | 15-18 | | NA | | NA | | 10.3  17.6 | | BW≤2500g  BW<10th centile (local) | | AFP, Method not reported  ≥90th centile | |
| Chitayat (88)  (2002) | | INC: singletons  Age not reported  (Canada) (case control, test) | | 1134 | | Second trimester | | NA | | NA | | 3.35 | | SGA no threshold | | DS≥1:385 and AFP≥2.2MoM  Method not reported | |
| Cho (89)  (1997) | | INC: singletons EXC: structural and chromosomal abnormalities  Mean 25.8 +/- 5.8 years  (USA) (case control, prospective, matched index test) | | 255 | | 14-20 | | NA | | NA | | 8.23 | | BW<10th centile | | AFP, RIA (Kallastaad)  ≤0.5, ≥2.5, 4.0 MoM | |
| Cox (90)  (1995) | | INC: singletons EXC: structural and chromosomal anomalies, invasive procedures, birth < 24 weeks  Age not reported  (Scotland) (cohort) | | 15705 | | 16-20 | | NA | | NA | | 2.29 | | BW<5th centile (local) | | AFP, RIA  >2.0 MoM | |
| Cusick (91)  (1996) | | INC: singletons, 47% primips, no fetoplacental abnormality EXC: structural and chromosomal anomalies, 2 placental abruption, TOP  Mean age 27.1 (15-42) years  (USA) (cohort, retrospective) | | 333 | | 15-20 | | NA | | NA | | 10.8 | | BW<10th centile (local, sex) | | AFP, Method not reported  ≥2.5 ≥3.0 MoM | |
| Di Mario (92)  (1998) | | INC: singletons EXC: structural and chromosomal anomalies, previous PET, IDDM, delivery < 26 weeks  Mean 30.6 +/- 3.6 years  (Italy) (cohort) | | 547 | | 16-18 | | NA | | NA | | 11.6 | | BW<10th centile (local) | | RIA (Johnson and Johnson)  AFP and HCG ≥2.0 MoM  UE3 ≤0.7MoM | |
| Doran (93)  (1987) | | INC: patients at low genetic risk, singleton  Age not reported  (Canada) (cohort, prospective) | | 7307 | | 16-18 | | NA | | NA | | 1.35 | | BW<10th centile | | AFP, RIA (WHO Behring)  >2.0 MoM | |
| Dugoff (66)  (2004) | | INC: singletons, 45.1% primips EXC: IDDM, structural and chromosomal anomalies  Mean age 30.1+/-5.77 years (16-53)  (USA) (cohort, prospective) | | 33995 | | 10+3-13+6 | | 2.3 | | BP>140/90 mmHg2x 6hrs apart, proteinuria >0.3g/24hrs or ≥2+ 2x 6hrs apart | | 8.80  3.82 | | BW<10th centile (local)  BW<5th centile (local) | | PAPP-A ELISA (Diagnostics, Texas)  ≤10th, <5th, ≤ 1st centile | |
| Dugoff (67)  (2005) | | EXC: structural and chromosomal anomalies  Mean age 30.2 +/- 5.71 (16-53) years  (USA) (cohort, prospective) | | 33145 | | 15-18+6 | | 2.23 | | Gestational hypertension with proteinuria (>0.3g/24hrs or ≥2+ 2x 6 hrs apart) | | 8.90  3.90 | | BW<10th centile (local)  BW≤5th centile | | AFP and HCG, Chemiluminescent immunoassay (Diagnostics)  ≥2.0 MoM  UE3 RIA (Diagnostics)  ≤0.5MoM  Inhibin A, ELISA (Serotec) ≥2.0MoM | |
| Dungan (94)  (1994) | | INC: singleton EXC: structural and chromosomal abnormalities, maternal age >35 years  (USA) (case control, matched test) | | 198 | | 15-20 | | NA | | NA | | 5.66 | | BW<5th centile | | DS≥1:270 | |
| Duric (95)  (2003) | | INC: singletons EXC: structural and chromosomal anomalies  Age <35 years  (Croatia) (cohort, retrospective) | | 673 | | 15-22 | | NA | | NA | | 5.20 | | BW<10th (local) | | RIA  AFP≥2.0 MoM  Total HCG ≥2.02MoM  UE3 ≤0.74MoM | |
| Endres (96)  (2003) | | INC: AFP>0.5 but <2.0 MoM, HCG≤0.5 MoM and estriol >0.6 and <2.0 MoM  Mean age 30+/-6 years  (USA) (case control matched) | | 438 | | 15-20 | | NA | | NA | | 7.30 | | BW<2500g | | ßHCG  Method not reported  ≤0.5 MoM | |
| Evans (97)  (1984) | | INC: screening programme  Age not reported  (UK) (case control, unmatched, index test) | | 220 | | 16-18 | | NA | | NA | | 7.73  3.18 | | BW<2500g  BW<10th centile | | AFP, RIA  (Amersham)  >95th centile | |
| Florio (35)  (2003) | | EXC: IDDM, chronic renal disease, SLE, APS, chronic hypertension.  Mean age 28.5+/-1.2 yrs  (Italy) (cohort) | | 58 | | 24 | | 31 | | BP≥140/90mmHg; proteinuria>0.3g/24hrs | | NA | | NA | | Inhibin A, immunoassay (Serotec UK)  >1.8MoM (140pg/ml) | |
| Ghosh (98)  (1986) | | EXC: structural and chromosomal anomalies  Age not reported  (Hong Kong) (cohort, prospective) | | 9838 | | 15-20 | | NA | | NA | | 3.03 | | SGA (no threshold) | | AFP, RIA  >2.0,≥2.8,3.0,4.0 MoM | |
| Gonen (99)  (1992) | | INC: HCG>2.5 MoM, singleton, USS dating EXC: structural and chromosomal anomalies, AFP>2.5 MoM  Age not reported  (Israel) (cohort) | | 493 | | 16-20 | | NA | | NA | | 7.91 | | BW<10th centile | | HCG, method not reported, (Delfia), >2.5MoM | |
| Gordon (100)  (1979) | | INC: singletons EXC: structural and chromosomal anomalies, delivery < 28 weeks  Age not reported  (UK) (cohort, prospective) | | 828 | | 16-22 | | NA | | NA | | 4.35 | | BW<2500g | | AFP, RIA  >95th centile | |
| Haddad (47)  (1999) | | INC: singleton, IVF, primips 86%  Mean age 33.6+/-4.2  (France) (cohort, retrospective) | | 180 | | 13-35 days | | 1.7 | | DBP>90mmHg 2x 24hrs proteinuria≥300mg/24hrs or ≥2+ 4hrs apart | | 10.60 | | BW<10th (local) | | HCG, Method not reported  <10th, >90th centile | |
| Haddow (101)  (1983) | | INC: singletons EXC: structural and chromosomal anomalies  Age not reported  (USA) (cohort, prospective) | | 2984 | | 15-20 | | NA | | NA | | 4.50 | | BW<2500g | | AFP, RIA (Oxford)  ≥2.0,3.0 MoM | |
| Haddow (102)  (1986) | | INC: singletons EXC: neural tube defects  Age not reported  (USA) (cohort) | | 6531 | | 15-20 | | NA | | NA | | 3.94 | | BW<2500g | | AFP, RIA  ≥2.0MoM | |
| Haddow (103)  (1987) | | INC: singletons EXC: structural and chromosomal anomalies  Age not reported  (USA) (cohort, prospective) | | 9507 | | 15-20 | | NA | | NA | | 4.08 | | BW<2500g | | AFP, RIA (Maine)  ≥2.0 MoM | |
| Hamilton (104)  (1985) | | INC: singletons EXC: structural and chromosomal anomalies  Age not reported  (Scotland) (case control, prospective, matched, index test) | | 372 | | 16-20 | | NA | | NA | | 15.90  17.70  4.30  9.95 | | BW<2500g  BW<10th centile (sex, local, parity)  BW<1500g  BW<5th centile (sex, local, parity) | | AFP, Method not reported  >2.5 MoM | |
| Hayashi (105)  (1992) | | INC: screening programme  Age not reported  (Japan) (cohort, prospective) | | 532 | | 12-19 | | NA | | NA | | 1.50 | | SGA (no threshold) | | AFP, Method not reported  ≥2.5 MoM | |
| Heikkila (106)  (2001) | | INC: singletons, primips, pre-eclampsia EXC: structural and chromosomal anomalies  Mean age 26.8+/-5.1 years  (Finland) (cohort, prospective) | | 487  471 | | 15-16 | | NA | | NA | | 2.22  2.95 | | BW<10th centile  BW<2500g | | HCG,  Immunoassay (Abbott)  ≥2.5MoM | |
| Heinonen (55)  (1996) | | INC: singletons EXC: structural and chromosomal anomalies, pregnancy loss < 24 weeks  Mean age not reported  (Finland) (case control, matched, test) | | 5290 | | 15 | | 4.6 | | Not reported | | 4.63 | | BW<2500g | | Total ßHCG  (IMX Abbott)  ≥2.0 MoM, >4 MoM | |
| Heinonen (107) (1999) | | INC: singletons EXC: structural and chromosomal anomalies, women that stopped smoking during study  Mean age 27.4 years  (Finland) (cohort) | | 1421 | | 15-18 | | NA | | NA | | 12.30  19.60 | | BW<2500g  BW<10th centile (sex) | | AFP< RIA (Clinical chemistry)  >2.5 MoM | |
| Hershkovitz (108)  (2003) | | EXC: structural and chromosomal anomalies  Mean age (AFP≥4.0MoM) 29.9+/-10.1 years  (Canada) (cohort, prospective) | | 121 | | 15-18 | | NA | | NA | | 3.31 | | BW<10th centile | | HCG, Method not reported  ≥4.0MoM | |
| Hershkovitz (69)  (2005) | | INC: chronic hypertension, previous PET, thrombophilia  Median age 29 (21-40)  (Canada) (cohort) | | 88 | | 15-18 | | 44.3 | | DBP≥90mmHg 2x ≥4 hrs apart, proteinuria >0.3g/24hrs or ≥2+ | | 26.1 | | BW<10th centile (sex) | | Method not reported  AFP >2.0 MoM  HCG ≥3.0MoM | |
| Jauniaux (53)  (1996) | | INC: singletons, abnormal uterine artery Doppler EXC: structural and chromosomal anomalies  Age not reported  (UK) (Cohort) | | 41 | | 20-24 | | 26.8 | | BP≥140/90mmHG persistent; proteinuria≥100mg/l | | 39.00 | | BW<10th centile | | AFP, FEIA (Hybritech)  ≥2.5 MoM  IRMA (Biomeriuex)  Total ßHCG>2.5MoM  Free ßHCG>2.5MoM | |
| Kavak (109)  (2006) | | INC: singletons, 50-4% primips EXC: IDDM, chronic hypertension, fetal abnormalities  Mean age 30.4+/-5 years  (Turkey) (cohort) | | 476 | | First trimester | | NA | | NA | | 7.35 | | BW<10th centile | | PAPP-A, Random access immunoassay (Kryptor)  <0.69MoM, <0.4 MoM(roc determined) | |
| Kiran (111)  (2005) | | INC: singletons, low risk EXC: structural and chromosomal anomalies  Mean age not reported.  (UK) (cohort) | | 6297 | | Second trimester | | NA | | NA | | 4.10 | | BW<2500g | | AFP, Method not reported >2.0 MoM | |
| Kowalczyk (75)  (1998) | | INC: singletons, 31.7% primips, AFP and HCG>2.0 MoM  EXC: structural and chromosomal anomalies  Age <35 years  (USA) (cohort) | | 309 | | 15-21 | | 4.2 | | SBP≥140mmHg or DBP≥90mmHg, proteinurai≥0.3g/24hrs or HELLP | | 8.74 | | BW<10th | | UE3, RIA  ≤0.75 MoM | |
| Krantz (112)  (2004) | | INC: first trimester screening EXC: chromosomal and structural anomalies  Age not reported  (USA) (cohort, retrospective) | | 6276 | | 10+4 – 13+6 | | NA | | NA | | 6.26 | | BW<10th centile  (ga, local, sex) | | Free ßHCG <1st and <5th centile, >90th and 99th centile  Papp-a <1st and 5th centile, >90th and 99th centile | |
| Kuo (113)  (2003) | | INC: singletons EXC: structural and chromosomal anomalies, abnormal HCG or Down’s risk, IDDM.  Mean age 28.3 +/-0.3 years  (Taiwan) (case control, unmatched, index test) | | 247 | | 15-20 | | NA | | NA | | 4.70  8.80 | | BW<2500g  BW<10th centile | | AFP, Method not reported  >2.0 MoM | |
| Kwik (114)  (2003) | | INC: singletons EXC: structural and chromosomal anomalies  Mean age 32.7 (15-42)  (Australia) (Cohort retrospective) | | 827 | | 77-97 days | | NA | | NA | | 6.65 | | BW<10th centile (local) | | PAPP-A ELISA (diagnostics)  <0.3,<0.5 MoM | |
| Lambert- Messerlian (44)  (2000) | | INC: singletons EXC: chronic hypertension, IDDM  Mean age 26.9+/-7.3 years  (USA) (case control, retrospective, outcome) | | 359 | | 15-21 | | Not reported | | BP>140/90mmHg, proteinuria>300mg/24hrs or ≥2+ | | NA | | NA | | Total HCG (Serono MAIO Clone) >2.3 MoM | |
| Lee (41)  (2000) | | INC: singletons, del > 24 weeks  Mean age 28.7+/-4.2 years  (Taiwan) (case control, retrospective, outcome) | | 1052 | | 15-20 | | 1.1  1.1 | | BP≥140/90mmHg 2x 6 hrs apart, proteinuria ≥1+  Severe PET SBP≥160mmHg or DBP≥110 mmHg 2x 6 hrs apart. Proteinuria ≥3+, oliguria | | NA | | NA | | ßHCG MEIA (Abbott) >2.0 MoM | |
| Legge (115)  (1985) | | NC: singletons EXC: structural and chromosomal anomalies  Age not reported  (New Zealand) (cohort) | | 507 | | 10-24 | | NA | | NA | | 8.68 | | BW<10th centile | | AFP, RIA (Biodata)  ≥2.0 MoM | |
| Lepage (116)  (2003) | | INC: MSAFP<2.0 MoM EXC: structural and chromosomal anomalies, IDDM  Age not reported  (Canada) (case control, matched, test) | | 2256 | | Second trimester | | NA | | NA | | 2.34 | | BW<10th centile | | HCG, Method not reported  ≥ 4.0 MoM | |
| Leung (42)  (2000) | | INC: singletons  Mean age 30.8+/-4.9 years  (China) (cohort) | | 1015 | | 18.1+/-1.3 | | 2.1 | | DBP≥90mmHg 2x 4hrs or ≥110mmHG 1x; prot.≥2+ 2x 4hrs apart or 300mg/24 hrs | | NA | | NA | | AFP MEIA (Imx Abbott) >2.0 MoM | |
| Lieppmann (117)  (1993) | | INC: singleton, downs risk >1:195 EXC: structural and chromosomal anomalies, women with normal HCG but raised AFP or estriol  Mean age not reported.  (USA) (cohort, prospective) | | 60 | | 15-18 | | NA | | NA | | 10.20  5.87 | | BW<10th centile (local)  BW<2500g | | HCG, RIA (MAIAClone Serono)  ≥ 2.0 MoM | |
| Luckas (50)  (1998) | | INC: primips EXC: multiple pregnancies, essential hypertension, IDDM, foetal abnormality  Age not reported  (UK) (cohort prospective) | | 430 | | 15-18 | | 4.4 | | Gestational/chronic hypertension, proteinuria≥300mg/24 hrs or ≥2+ or with HELLP; Davey and MacGillvray 1988 | | NA | | NA | | HCG RIA (Amerlex-M)  >2.0 MoM | |
| Markestaad (118)  (1997) | | INC: multips  Mean age if SGA 28.8+/-0.4 years, non-SGA 30.2+/-0.4 years  (USA) (cohort, prospective) | | 216 | | <20 | | NA | | NA | | 47.22 | | BW<15th centile (sex, parity, local) | | HCG, Immunoreactive (Seano)  <10th centile  UE3, RIA (Amersham) <10th centile | |
| Milunsky (60)  (1989) | | INC: singletons  20-34 years (90%)  (USA) (cohort) | | 13486 | | 15-20 | | 1.7 | | Not reported | | 2.28 | | BW<5.5 pounds | | AFP, RIA (Clinical assays)  ≥2.0 MoM, ≤0.4MoM | |
| Milunsky (119)  (1996) | | INC: singletons EXC:IDDM, structural and chromosomal anomalies  Mean age 30.3 years  (USA) (case control matched test) | | 78 | | 15-24 | | NA | | NA | | 10.20 | | BW<2500g | | DS≥1:270 | |
| Miyakoshi (120)  (2001) | | INC: singletons, primips 70% EXC: structural and chromosomal anomalies  Mean age 38.9+/-1.8  (Japan) (cohort, retrospective) | | 359 | | 15-18 | | NA | | NA | | 11.42 | | BW<10th centile | | HCG, Method not reported  >2.0 MoM | |
| Morssink (121)  (1995) | | INC: singletons EXC: structural and chromosomal anomalies, IDDM, delivery < 28 weeks  Age not reported  (Netherlands) (cohort) | | 8892 | | 15-20 | | NA | | NA | | 10.10 | | BW<10th centile (local) | | AFP and HCG, EIA (Abbott)  >2.5 MoM | |
| Morssink (62)  (1997) | | INC: singletons EXC: IDDM, structural or chromosomal anomalies  Mean age 28 years  (Netherlands) (cohort) | | 2008 | | 15-20 | | 2.0 | | Diastolic rise >15mmHG; Proteinuria≥300mg/24 hrs; Davey and MacGillivray 1988 | | NA | | NA | | AFP and HCG, method not reported >2.5 MoM | |
| Muller (56)  (1996) | | INC: normotensive, PIH, SGA  Mean age not reported  (France) (cohort) | | 5776 | | 15-18 | | 0.6 | | SBP≥140mmHg or DBP≥90mmHg 2x 10 hrs apart, proteinuria>300mg/l | | NA | | NA | | HCG EIA >2.0 MoM | |
| Muttukrishna (43)  (2000) | | EXC: chronic hypertension, aspirin therapy, multiple pregnancy.  Mean age 27.6+/-4.3  (UK) (case control, retrospective, outcome) | | 297 | | 15-19 | | 4.8 | | SBP>140 or DBP>90mmHg twice (or increase 30/15 mmHg)  Proteinuria>0.3g/24hrs or >1g/l on spot testing | | NA | | NA | | Inhibin A, ELISA (Life) >90th centile | |
| Mwambingu (122)  (1985) | | INC: singletons EXC: structural and chromosomal anomalies  Age not reported  (Scotland) (cohort) | | 282 | | 16-18 | | NA | | NA | | 13.48 | | SGA (no threshold) | | AFP, Method not reported  >2.5 MoM or > 97th centile | |
| Naylor (123)  (2001) | | INC: singletons EXC: IDDM, hypertension, maternal illnesses associated with adverse pregnancy outcome  Mean age 27.8+/-7.7 years  (USA) (case control, prospective) | | 150 | | 15-24 | | NA | | NA | | 5.33 | | BW<10th centile | | DS>1:190 | |
| Odibo (124)  (2006) | | INC: singletons EXC: structural and chromosomal anomalies  Mean age 25.8 +/-7.0  (USA) (case control, retrospective, outcome) | | 2040 | | Second trimester | | NA | | NA | | 12.50 | | BW<5th centile (local) | | Method not reported  AFP>2.0 MoM  HCG >2.5 MoM  UE3≤0.9MoM | |
| Ogle (125)  (2000) | | INC: singletons, 53% primips EXC: structural and chromosomal anomalies  Mean age not reported  (UK) ( case control index test, nested cohort) | | 544 | | 15-18 | | NA | | NA | | 3.31 | | IUGR (threshold not reported) | | DS>1:270  AFP MEIA  Free βHCG ELISA | |
| Onderoglu (51)  (1997) | | INC: singletons EXC: IDDM, structural and chromosomal anomalies, MSAFP >2.0MoM, raised AFP and HCG  Mean age 30.1+/-5.2 years  (Turkey) (case control, nested) | | 562 | | 15-20 | | 2.7 | | BP rise 30/15mmHg over 1st trimester values or persistent BP≥140/90mmHg, proteinuria≥500mg/l | | 3.56 | | BW<10th centile | | HCG, Dunzen method  >2.0 MoM | |
| Ong (9)  (2000) | | INC: singletons,32.0% primips  Mean age 29.2 (15-45)  (UK) (cohort, retrospective) | | 5297 | | 10-14 | | 2.5 | | DBP≥90mmHg twice 4 hrs apart or ≥110mmHg once; proteinuria ≥1+ or >0.3g/24hrs | | 7.46  3.23 | | BW<10th centile  BW<5th centile | | Free ßHCG and PAPP-A, random access immunoassay  (Kryptor)  <5th, <10th centile and <median | |
| Pergament (126)  (1995) | | INC: singletons, age <35 years, amniocentesis EXC: structural and chromosomal anomalies  Mean age cases 30.0+/-3.8, controls 30.0+/-3.7 years  (USA) (cohort, retrospective) | | 174 | | 15-20 | | NA | | NA | | 1.72 | | BW<10th centile | | DS>1:250  All RIA | |
| Pilalis (74)  (2007) | | INC: singletons, Papp-a, TVS uterine artery Doppler, known outcome EXC: 4 miscarriages, 11 terminations  Mean age 29 (15-45)  (Greece) (cohort, prospective) | | 878 | | 11-14 | | 1.5 | | SBP>140 or DBP>90nnHg twice 6 hrs apart; proteinuria>0.3g/24hrs or ≥2+ dipstix | | 10.7  4.00 | | BW<10th centile  BW<5th centile | | PAPP-A, immunoassay (Kryptor)  ≤5th and <10th centile | |
| Pouta (49)  (1998) | | INC: nulliparas EXC: multiple pregnancies, foetal defects  Mean age 27.7 +/- 4.5 years  (Finland) (cohort) | | 637 | | 15-19 | | 5.3 | | BP≥140/90mmHg 2x 6hrs apart or rise 30/15mmHg;proteinuria ≥300mg/24 hours | | NA | | NA | | AFP and ßHCG (Wallace) >2.0MoM | |
| Raty (48)  (1999) | | Mean age 26.9+/-3.6 years  (Finland) (case control retrospective, outcome) | | 1242 | | 15.9+/-1.2 | | 4.9  0.8(severe) | | Severe PET:BP≥16-/110mmHg; proteinuria≥5.0g/l, oliguria, subjective symptoms | | NA | | NA | | AFP IFMA (Wallace) >2.0 MoM | |
| Roes (63)  (2004) | | Not reported  (Netherlands) (case control) | | 55 | | 6-15 | | 34.5 | | DBP≥90mmHg twice >4 hours apart, proteinuria >0.3g/l | | NA | | NA | | Inhibin A, ELISA, >366pg/ml or >438pg/ml | |
| Roiz-Hernandez (70)  (2006) | | INC: singletons EXC: IDDM, chronic hypertension.  Mean age not reported.  (Mexico) (cohort, prospective) | | 784 | | 16-21 | | 6.9 | | DBP≥90mmHg twice >4 hours apart, proteinuria >0.3g/l | | NA | | NA | | HCG, Abbott IMX>2.0MoM | |
| Roop (127)  (1991) | | EXC: lost to follow up  Mean age 27.7 years  (USA) (Cohort) | | 1703 | | 15-20 | | NA | | NA | | 3.23 | | IUGR (no threshold) | | AFP, RIA (Clinical assays)  >2.3 MoM | |
| Sebire (45)  (2000) | | INC: singletons  Median age 34 (16-35)  (UK) (cohort) | | 759 | | 10-14 | | 1.2 | | BP>140/90mmHg twice 6hrs apart; proteinuria≥1+ or >0.3g/24hrs | | NA | | NA | | Inhibin A, ELISA, >95th centile | |
| Secher (128)  (1985) | | INC: singletons, primips, birth > 28 weeks EXC: neural tube defects  Age not reported  (Denmark) (cohort) | | 1739 | | 16-18 | | NA | | NA | | 10.60  5.29 | | BW<10th centile (local)  BW<5th centile (local) | | AFP, RIA  >1.0, 1.5, 2.0 MoM | |
| Simpson (59)  (1995) | | INC: singletons EXC: structural and chromosomal anomalies  Age not reported  (USA) (cohort) | | 650 | | 15-20 | | 9.7 | | SBP≥140mmHg or DBP≥90mmHg 2x; prot≥1+ 2x or ≥300mg/24 hours | | 10.50  2.77 | | BW<2500g  BW<10th | | AFP, EIA (Hybritech Tandem ERA, Abbott)  ≥2.0 MoM | |
| Smith (39)  (2002) | | INC: singletons, primips 44.4%, EXC: structural and chromosomal anomalies  Median age 30.7 years  (UK) (cohort, prospective) | | 8839 | | 8-14 | | 3.74 | | PIH with proteinuria | | 4.18 | | BW<5th centile | | Free ßHCG and PAPP-A, random access immunoassay  (Kryptor)  <5th centile | |
| Smith (129)  (2006) | | INC: singletons, screening programme, birth ≥24 weeks  Median age 29 (25-33)  (UK) (cohort, prospective) | | 8483 | | 15-21 | | NA | | NA | | 4.16 | | SGA (no threshold) | | AFP, Method not reported  ≥1.7 MoM (97th centile)  PAPP-A, method not reported <5th centile | |
| Spencer (71)  (2005) | | INC: singleton, all screened women.  Median 30 (16-47)  (UK) (cohort, prospective) | | 4063 | | 11-13+6 | | 1.46 | | ISSHP guidelines 2001; proteinuria ≥0.3g/24hrs | | NA | | NA | | HCG (Kryptor) <5th centile  PAPP-A, ELISA, ≤5th centile (0.422 MoM) | |
| Spencer (72)  (2006) | | EXC: chronic renal disease  Mean age not reported  (UK) (case control, retrospective, outcome) | | 168 | | 22-24+6 | | 14.3 | | DBP>90mmHg twice 4hours apart or >110 mmHg; proteinuria ≥1+ or >0.3g/24hrs | | NA | | NA | | Papp-a (Kryptor), 5% or 10% FPR  HCG (Kryptor), 5% or 10% FPR  Inhibin A, ELISA, Serotec, 5% or 10% FPR | |
| Sritippayawan (130)  (2005) | | INC: singletons EXC: uninterpretable results, delivery at another hospital, bad obstetric or past medical or family history, structural or chromosomal anomalies  Mean age cases 34.5(6.4) , controls 33.7 (5.2) years  (Thailand) (case control, matched test) | | 330 | | 14-21 | |  | |  | | 1.21  5.15 | | BW<10th centile  BW<2500g | | DS>1:270  AFP – RIA HCG - EIA | |
| Stamilio (46)  (2000) | | INC: non smokers, mild PET in control group EXC: multiple pregnancies, foetal anomalies  Mean age 25.7+/-0.3 years  (USA) (cohort) | | 1998 | | 15-19 | | 6.4  (2.5) severe | | Severe PET: SBP≥160mmHg or DBP≥110mmHg;proeinuriat ≥3+ or ≥5.0g/24hrs (oliguria, symptoms) | | NA | | NA | | AFP and HCG not reported >2.0 MoM  UE3 method not reported ≤0.9MoM | |
| Summers (131)  (2003) | | INC: singletons EXC: IDDM, structural and chromosomal anomalies, positive NTD screen  Median age 34 years  (Canada) (case control, retrospective, nested cohort, index test) | | 23098 | | 115 days median | | NA | | NA | | 1.68 | | SGA (no threshold) | | DS>1:385 | |
| Tanaka (132)  (1994) | | INC: singletons  Age not reported  (Japan) (cohort) | | 1097 | | 15-18 | | NA | | NA | | 10.00 | | BW<2500g | | AFP RIA, HCG TRFIA  ≥2.0 MoM | |
| Towner (133)  (2006) | | INC: screened, singleton  EXC: pregnancy loss <20 weeks  Mean age 26.7 +/-6  (USA) (case control, matched, test) | | 618 | | <20 weeks | | NA | | NA | | 8.41 | | BW<10th centile (ga, local, sex) | | HCG, method not reported  ≥2.0 MoM | |
| Tul (134)  (2003) | | INC: singletons, 51% primips  Mean age 30.4 (18-44) years  (Slovenia) (cohort, retrospective) | | 1004 | | 10-14 | | NA | | NA | | 5.07 | | BW<10th centile (local) | | PAPP-A, Random access immunoassay (Kryptor) ≤0.5MoM | |
| Vaillant (58)  (1996) | | EXC: multiple pregnancies, Down’s syndrome, IVF  Mean age 29+/-5 years  (France) (cohort) | | 434 | | 14-20 | | 3.7 | | DBP≥90mmHg 2x 24 hrs apart or DBP≥100mmHg>22 weeks, proteinuria>300mg/24hrs or ≥2+ | | NA | | NA | | ßHCG EIA >41000 IU | |
| Wald (135)  (1977) | | INC: singletons EXC: structural and chromosomal anomalies  Mean age not reported  (UK) (case control, prospective, matched, index test) | | 188 | | 4-22 | | NA | | NA | | 9.04 | | BW<2500g | | AFP, RIA  ≥ 3.0 MoM | |
| Wald (136)  (1980) | | INC: singletons EXC: structural and chromosomal anomalies  Mean age not reported  (UK) (cohort) | | 4198 | | 16-18 | | NA | | NA | | 5.40 | | BW≤2500g | | AFP, Method not reported  > 2.0 MoM | |
| Wald (73)  (2006) | | Not reported.  Mean age not reported  (UK) (nested case control, matched) | | 576 | | 15-20 | | 16.7 | | DBP>90mmHg twice a day apart; proteinuria ≥2+ or >0.5g/24hrs | | NA | | NA | | AFP, free βHCG, estriol, PAPP-A, Inhibin A, triple test, quadruple test (5,10,15% FPR) | |
| Waller (54)  (1996) | | INC: singletons EXC: structural and chromosomal anomalies  Mean age 27 years  (USA) (cohort, retrospective) | | 51008 | | 15-19 | | 1.3 | | Not reported | | 5.16 | | BW<5th centile | | AFP, EIA (Abbott)  >1.0,2.0,2.5 MoM  <0.44 MoM (1st centile) | |
| Weiner (137)  (1991) | | INC: singletons, referred for amniocentesis EXC: structural and chromosomal anomalies, TOP, miscarriage  Mean age 32.7 +/-6.0  (USA) (cohort) | | 144 | | Second trimester | | NA | | NA | | 6.94 | | BW<10th centile | | AFP, RIA (Amersham)  >2.0 MoM | |
| Wenstrom (138)  (1992) | | INC: singletons, screened with raised AFP and repeat sample EXC: structural and chromosomal anomalies  Age not reported  (USA) (Cohort) | | 440 | | 15-20 | |  | |  | | 12.0 | | SGA no threshold | | AFP, method not reported  ≥2.5 MoM | |
| Wenstrom (57)  (1996) | | INC: singletons EXC: raised AFP or acetylcholinesterase in amniotic fluid, blood contamination of amniotic fluid, structural or chromosomal anomalies  Age not reported  (USA) (cohort, retrospective) | | 4336(FGR)  4614 (PET) | | Second trimester | | 1.1 | | Not reported | | 3.48 | | BW<10th (local) | | AFP< RIA (Sanofi Pasteur)  ≥2.5 MoM | |
| Westergaard (139)  (1984) | | INC: singletons  Age not reported  (Denmark) (cohort prospective) | | 208 | | 18-22 | | NA | | NA | | 15.9 | | BW<10th centile (local) and phenotypic signs of FGR | | PAPP-A, EIA, <10th centile | |
| Williams (140)  (1992) | | INC: singletons EXC: structural and chromosomal anomalies  Mean age 28.8 +/- 4.5  (USA) (case control, prospective, unmatched test) | | 412 | | Second trimester | | NA | | NA | | 18.70  14.3 | | BW<2500g  BW<10th centile (local) | | AFP, EIA (Hybritech)  ≥2.0 MoM | |
| Yaron (7)  (1999) | | EXC: structural and chromosomal anomalies  Age not reported  (USA) (cohort) | | 60040  45565  24504  20907 | | 14-22 | | 3.2 | | SBP≥140mmHg or DBP≥90mmHg; presence of proteinuria | | 2.47  2.32  4.93  1.76 | | BW<5th centile | | AFP, RIA (Sanofi)  >2.5 MoM  ßHCG IRMA (Biodata)  >2.5 MoM  UE3 Competitive immunoasay  <0.5 MoM  Triple test | |
| Yaron (37)  (2002) | | INC: singletons  EXC: structural and chromosomal anomalies  Mean age 30.4+/-4.3 years  (USA) (cohort) | | 1622 | | 10-13 | | 1.7 | | DBP≥110mmHg 1x or ≥90mmHg 2x 4 hrs apart, proteinuria>300mg/24 hrs or >1+ | | 3.02 | | BW<5th centile | | Free ßHCG FIA (Delfia Wallace)  >5.0,4.0,3.0,2.0,1.0 MoM | |
| Yaron (38)  (2002) | | INC: singletons  Mean age not reported  (USA) (cohort) | | 1622 | | 10-13 | | 1.7 | | DBP≥110mmHg 1x or ≥90mmHg 2x 4 hrs apart, proteinuria>0.3g/24hrs or ≥2+ | | NA | | NA | | PAPP-A, fluorimmunassay, <0.25, <0.5, <0.75, <1 MoM | |
| Yuong Kim (110)  (2000) | | INC: singletons EXC: IDDM, HCG>2MoM, AFP>2MoM  Mean age 29.0+/-2.6 years  (Korea) (cohort) | | 1096 | | 15-21 | | NA | | NA | | 3.65 | | BW<10th centile | | UE3, Method not reported ≤0.75 MoM | |
| Zarzour (141)  (1998) | | INC: amniocentesis EXC: abdominal wall defects  Age not reported  (USA) (cohort, retrospective) | | 1904 | | 14-20 | | NA | | NA | | 8.25 | | BW<2500g | | AFP, RIA  >2.0 MoM | |
| Zeeman (36)  (2003) | | INC: chronic hypertension  Mean age 33.6+/-5.5 and 31.6+/-7.2  (USA) (cohort) | | 61 | | 16-28 | | 34.4 | | Worsening hypertension, proteinuria ≥1+ or >0.3g/24hrs | | NA | | NA | | Inhibin A, ELISA (Serotec), > mean +2sd | |
|  |  | |  | |  | |  | |  | |  | |  | |  | |  |

FPR false positive rate; SBP systolic blood pressure; DBP diastolic blood pressure, prot. Proteinuria; hrs hour; INC inclusion; EXC exclusion; AFP alpha-fetoprotein; HCG human chorionic gonadotrophin;UE3 unconjugated estriol; PAPP-A pregnancy associated plasma protein A; PET preeclampsia; PIH pregnancy induced hypertension; FGR fetal growth restriction; CH chronic hypertension; GH gestational hypertension; IDDM diabetes mellitus; AID auto immune disease; APS antiphospholipid syndrome; SLE systemic lupus erythematodes; MoM multiples of the median. TP true positives; BW birth weight; TVS transvaginal; UK United Kingdom; USA United States of America; NA not applicable; RIA random access immunoassay; ELISA enzyme linked immunoabsorbent assay; EIA enzyme immunoassay; FEIA fluroenzyme immunoassay; IRMA immunoradiometric assay; MEIA microparticle enzyme immunoassay; IFMA immunoflurometric assay; TRFIA time resolved flurometricimmuno assay; mg milligrams; mmmHg millimetres of mercury; µg/l mircrograms per litre; pg/ml pictograms per millilitre; g grams; NT nuchal translucency; ROC receiver operating characteristic curve; SGA small for gestational age; NTD neural tube defects; MSAFP maternal serum alpha feto-protein; BP blood pressure; IUGR intrauterine growth restriction; DS Down’s syndrome; TOP termination of pregnancy; USS ultrasound scan; IVF in-vitro fertilisation; HELLP haemolysis elevated liver enzymes low platelets syndrome; ISSHP International Society for the Study of Hypertension in Pregnancy; ga gestational age; sd standard deviation, % percent..
