# Supplementary material for: Serum screening with Down's syndrome markers to predict pre-eclampsia and small for gestational age: Systematic review and meta-analysis
Source: BMC Pregnancy Childbirth. 2008 Aug 4;8:33. doi: 10.1186/1471-2393-8-33 (PMC2533288; doi:10.1186/1471-2393-8-33)
Supplement: Additional file 6 — "Forest plots of sensitivity and specificity." Results of sensitivity and specificity displayed as Forest plots. [file 1471-2393-8-33-S6.doc]

Figure 1: Forest Plot showing sensitivity and specificity with 95% confidence intervals (95% CI) for studies of alpha feto-protein (AFP) to predict pre-eclampsia and small for gestational age (birth weight threshold as indicated). Results with diamonds are pooled results (number of studies as indicated), results with squares are single studies. The number of women included in the studies is shown, all studies second trimester.

Figure 2: Forest Plot showing sensitivity and specificity with 95% confidence intervals (95% CI) for studies of human chorionic gonadotrophin (HCG) to predict pre-eclampsia and small for gestational age (birth weight threshold as indicated). Results with diamonds are pooled results (number of studies as indicated), results with squares are single studies. The number of women included in the studies is shown,( ^a^  first trimester testing).

Figure 3: Forest Plot showing sensitivity and specificity with 95% confidence intervals (95% CI) for studies of unconjugated estriol to predict pre-eclampsia and small for gestational age (birth weight threshold as indicated). Results with diamonds are pooled results (number of studies as indicated), results with squares are single studies. The number of women included in the studies is shown, all second trimester testing.

Figure 4: Forest Plot showing sensitivity and specificity with 95% confidence intervals (95% CI) for studies of pregnancy associated plasma protein A (PAPP-A) to predict pre-eclampsia and small for gestational age (birth weight threshold as indicated). Results with diamonds are pooled results (number of studies as indicated), results with squares are single studies. The number of women included in the studies is shown, (^a^ first trimester testing).

Figure 5: Forest Plot showing sensitivity and specificity with 95% confidence intervals (95% CI) for studies of inhibin A to predict pre-eclampsia and small for gestational age (birth weight threshold as indicated). Results with diamonds are pooled results (number of studies as indicated), results with squares are single studies. The number of women included in the studies is shown, (^a^ first trimester testing).
