# Supplementary figures and images for: Serum screening with Down's syndrome markers to predict pre-eclampsia and small for gestational age: Systematic review and meta-analysis
Source: BMC Pregnancy Childbirth. 2008 Aug 4;8:33. doi: 10.1186/1471-2393-8-33 (PMC2533288; doi:10.1186/1471-2393-8-33)

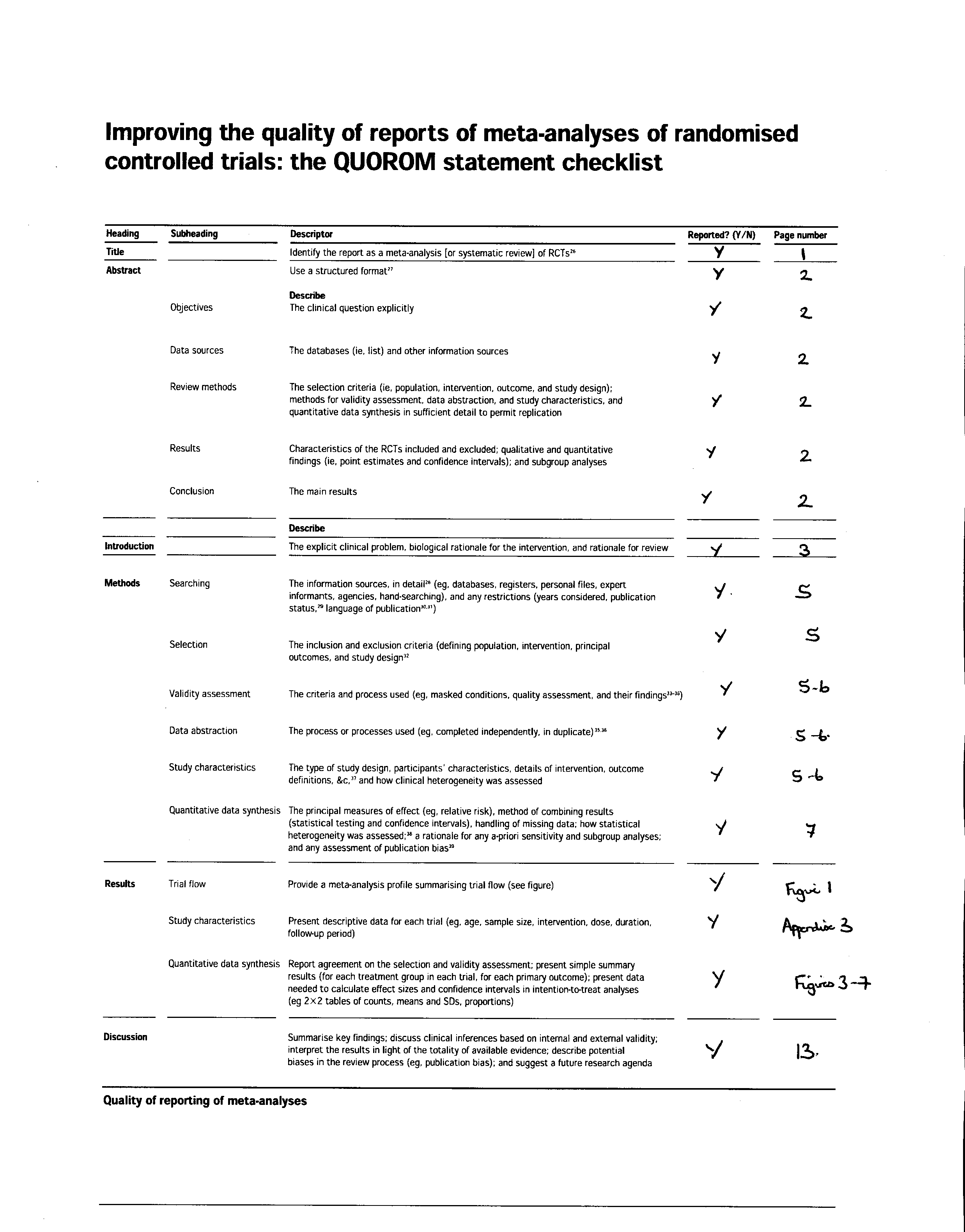

Supplement: Additional file 7 — "Improving the quality of reports of meta-analyses of randomised controlled trials: the Quorum Statement checklist." The Quorum statement checklist. [file 1471-2393-8-33-S7.bmp]
